# A multidisciplinary RNA-guided approach to complement genomic analysis of unsolved patients with an inborn error of immunity

**DOI:** 10.3389/fimmu.2026.1829883

**Published:** 2026-05-28

**Authors:** Willem T. K. Maassen, Lotte C. E. T. Pape, Tim Niemeijer, Anne-Margriet Heijink, Martine T. Meems-Veldhuis, Daniëlle J. Boerrigter, Gerben van der Vries, Helga Westers, Lennart F. Johansson, Kasper J. van der Velde, Morris A. Swertz, Lude Franke, Geertje E. Legger, Annechien J. A. Lambeck, Abraham Rutgers, Iris H. Jonkers, Mariëlle E. van Gijn, Evelien Zonneveld-Huijssoon

**Affiliations:** 1 Genomics Coordination Center, University of Groningen, University Medical Center Groningen, Groningen, Netherlands; 2 Department of Genetics, University of Groningen, University Medical Center Groningen, Groningen, Netherlands; 3 Department of Paediatrics, University of Groningen, University Medical Center Groningen, Groningen, Netherlands; 4 Department of Medical Immunology, University of Groningen, University Medical Center Groningen, Groningen, Netherlands; 5 Department of Rheumatology and Clinical Immunology, University of Groningen, University Medical Center Groningen, Groningen, Netherlands; 6 Genome Analysis Laboratory, Department of Human Genetics, Amsterdam University Medical Center, Amsterdam, Netherlands

**Keywords:** aberrant gene expression, aberrant splicing, diagnostics, inborn errors of immunity, mono-allelic expression, multidisciplinary team, RNA-sequencing

## Abstract

**Introduction:**

Inborn errors of immunity (IEI) comprise a heterogeneous, and growing, group of over 550 disorders linked to over 500 genes. Current diagnostic rates for IEI range from 15–70%, with missed diagnoses likely explained by variants of uncertain significance that lack evidence for reclassification and/or variants undetectable with current methods. To overcome these limitations, we developed a structured, RNA-guided approach to reanalyze unsolved IEI patients and increase the diagnostic yield of genetic testing.

**Methods:**

In a multidisciplinary team, we analyzed a cohort of 22 patients suspected to have an IEI for whom standard diagnostic genetic testing was inconclusive. We systematically evaluated whether aberrant expression, aberrant splicing or mono-allelic expression, based on the detection of expression outliers, splicing outliers and allele-specific read counts at heterozygous single nucleotide variants could reveal potentially causative variants that aligned with the clinical phenotype and expected mode of inheritance.

**Results:**

In one male patient, we detected a splice variant in *IKBKG* (NM_001099857.5: c.671 + 2T>G) that causes exon 5 skipping, which explains his phenotype. In one female patient, we detected a pathogenic splice variant in the X-linked recessive gene *CYBB* (NM_000397.4: c.45 + 5G>A) that, in combination with skewed X-inactivation, caused a significant decrease in functional transcripts. We also detected a deep-intronic variant (NM_003998.4: c.1495 + 506T>C) that activates a cryptic splice site, leading to a pseudo-exon in *NFKB1* in a patient with a phenotype consistent with *NFKB1* haploinsufficiency.

**Conclusion:**

We could provide a conclusive diagnosis for 2 out of 22 patients, underscoring how RNA-guided variant interpretation can improve genetic diagnostic yield in IEI patients. With advances in interpretation technologies, integrating RNA-sequencing into routine diagnostics could be a pivotal step toward achieving more comprehensive and precise genetic diagnoses of IEI.

## Introduction

The inborn errors of immunity (IEI) are a constantly growing, heterogenous group of 559 disorders caused by variants in 508 different genes (1, 2). Although each individual IEI is rare, research from the European Society for Immunodeficiencies (ESID) estimates the prevalence of IEI in the Netherlands to be 3.96 in 100,000 (3). IEI can be categorized into subtypes ranging from susceptibility to infections (primary immunodeficiencies (PID)) to an overactivation of the inflammatory response (systemic autoinflammatory diseases). The importance of early diagnosis for IEI is made clear by the decrease in the percentage of patients experiencing infections post-diagnosis (4), and early diagnosis can reduce reliance on unnecessary (invasive) diagnostic procedures and avoid inappropriate therapeutic strategies. This is critical, as prolonged inflammation and persistent infection substantially increase the risk of complications, including systemic amyloidosis. Nevertheless, clinical overlap between various IEI continues to complicate clinical diagnosis and appropriate treatment (4).

Genetic testing has been shown to support early diagnosis and to improve disease management in 76% of patients with a genetic diagnosis (5). Current genetic testing for IEI primarily relies on exome sequencing (ES), which targets regions in and close to exons of protein-coding genes. Using IEI-specific gene panels, this achieves a diagnostic yield of approximately 38% (range 15–70%) (6). This might be because 20–50% of detected variants remain classified as variants of uncertain significance (VUSs) (7, 8). Additionally, causal variants may go undetected, for example those located in non-coding regions or in genes that have not yet been linked to IEI. Unlike ES, genome sequencing (GS) captures both coding and non-coding regions, but GS appears to only marginally increase the diagnostic rate, possibly due to the increase in interpretative burden and challenges in the interpretation of non-coding variants (9, 10).

In recent years, RNA-sequencing (RNA-seq) has gained traction as a diagnostic tool by revealing the functional effects of genetic variants on gene expression and splicing, enabling more selective interpretation. Multiple studies have demonstrated that incorporating patient-level analyses of aberrant expression, aberrant splicing and mono-allelic expression can increase diagnostic yield in general by 4–12% (11–15). For IEI specifically, depending on the subtype, up to 90% of IEI-related genes are sufficiently expressed in whole blood (WB) (16). This makes WB a minimally invasive and clinically relevant tissue for detecting immune-related molecular defects. We therefore argue that RNA-seq offers a pragmatic and immediately actionable approach to detect affected genes in suspected IEI cases that go undiagnosed in GS. This applies to ES as well, potentially unravelling the unknown functional consequences of >75% variants in the ~19,500 genes (17, 18). Therefore, RNA-seq offers valuable complementary insight to diagnose a wide spectrum of diseases in addition to IEI.

In this study, we show the added value of a structured RNA-guided approach for molecular diagnosis of IEI patients, using the MOLGENIS Variant Interpretation Pipeline (VIP) (19), OUTRIDER (20), FRASER (21) and the tMAE module from DROP (22). While different methods to detect gene expression outliers and outlier usage of splice junctions exist (23–26) (from here on out we will refer both types of outliers as expression outliers and splicing outliers, as adopted form the authors of OUTRIDER and FRASER), we used OUTRIDER and FRASER as they are specifically designed for rare disease diagnostics. They provide a framework to compare single patients with the remainder of a cohort using built-in methods to automatically detect and correct for noise and known and unknown batch effects in bulk RNA-sequencing data. This is especially useful for studying heterogeneous datasets containing rare IEI cases. They have been widely used in different studies (11, 14–16, 27). Some of these studies have also shown successful correction of hidden batch effects and successful aberrant gene expression and aberrant splicing detection using whole blood, a clinically relevant tissue for IEI (11, 16, 27). Aided by new insights from the RNA-seq data, a multidisciplinary team reanalyzed the ES data of a cohort of 22 clinically diagnosed IEI patients without a molecular diagnosis after routine genetic diagnostics at the University Medical Center Groningen (UMCG). Using this approach, we aim to demonstrate that RNA-seq can uncover functional insights in IEI and point to genetic variants that would otherwise remain undetected or appear insignificant when using standard−of−care diagnostic methods.

## Materials and Methods

### Patient cohort

**Figure 1:**
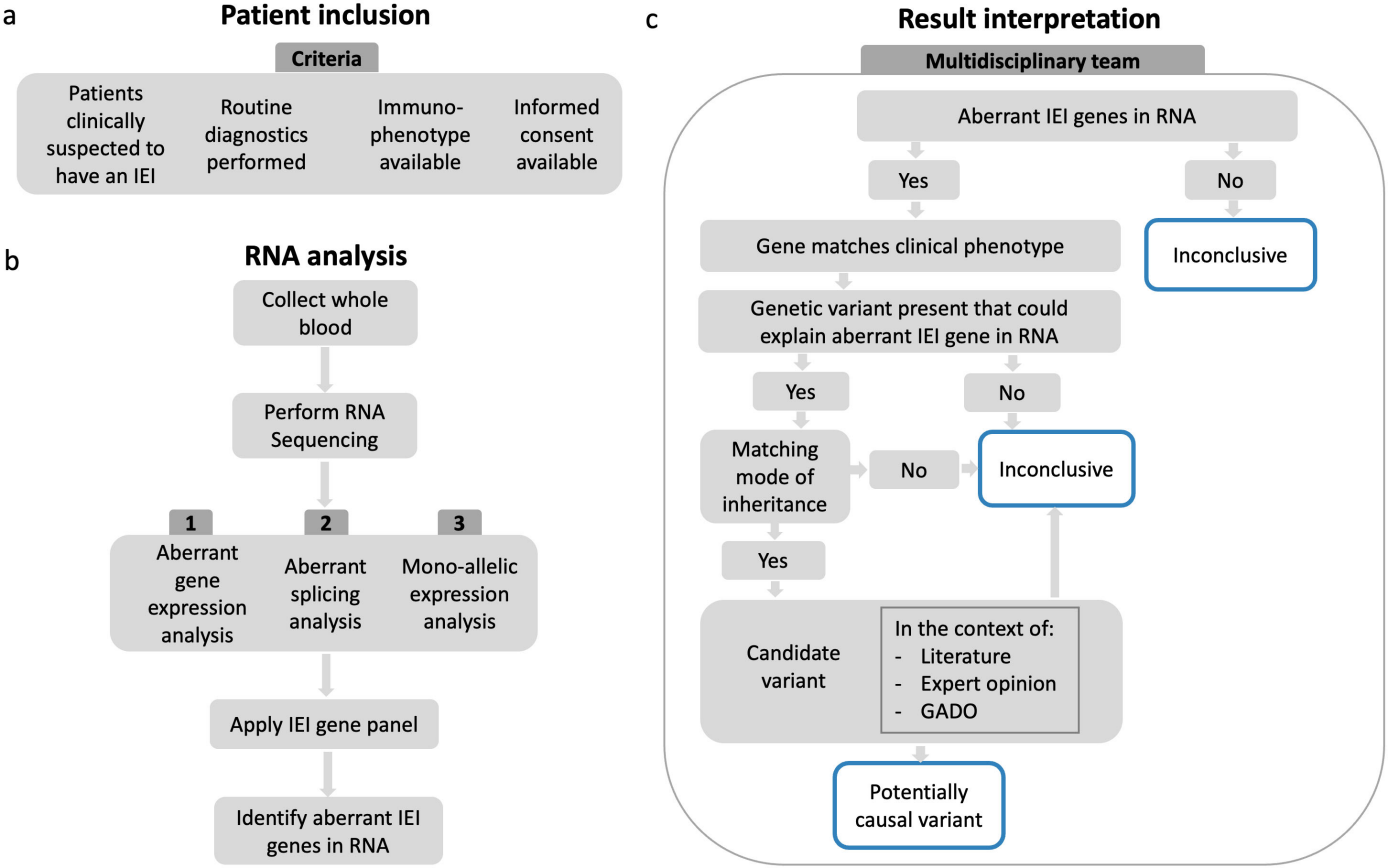
Study design. **(A)** All included patients were clinically suspected to have an IEI, and routine diagnostics were performed on DNA isolated from whole blood (WB) using IEI gene panel analysis on WES data. All patients were also immunophenotyped and provided informed consent to participate in the study. **(B)** For this study, we collected WB in PAXgene tubes and performed aberrant gene expression, aberrant splicing and mono-allelic expression analysis to identify aberrant genes within the IEI gene panel. **(C)** Aberrant genes within the IEI gene panel were filtered based on their match with the clinical phenotype, whether they contained a variant that could cause the aberrant effect in RNA and the gene’s mode of inheritance. Candidate variants could be interpreted as potentially causal based on existing literature in the Online Mendelian Inheritance in Man (OMIM) database, expert opinions and extended gene network search using GADO.

Patients were recruited at the Department of Genetics of the University Medical Center Groningen (UMCG) between June 2022 and September 2025. All individuals had previously undergone routine clinical genetic testing, including ES, which did not yield a definitive molecular diagnosis. Next, each case was discussed in a multidisciplinary team (MDT) consisting of clinical immunologists, clinical geneticists and molecular specialists. During the MDT review, eligibility for inclusion in the present study was evaluated. Inclusion criteria required a strong clinical suspicion of a monogenic IEI despite the absence of a genetic diagnosis through standard diagnostic procedures (**Supplementary Data Sheet 1**). Only patients who met this criterion and lacked a conclusive molecular finding after ES were eligible for inclusion (**Figure 1A**). In total, 22 patients were recruited (11 male and 11 female) with ages ranging from 3 months to 61 years.

DNA isolation was performed at the UMCG and library prep and sequencing was performed at GenomeScan (GenomeScan B.V., Leiden, the Netherlands). DNA was isolated from WB of all patients and processed using the Agilent SureSelect XT and Human All Exon V7 library preparation kits (Agilent Technologies, Inc., Santa Clara, CA, USA). ES was performed on the NovaSeq 6000 platform (Illumina, Inc., San Diego, CA, USA). The original Variant Call Format (VCF) files were generated using our in-house NGS-DNA pipeline for variant analysis, using reference genome build GRCh37 and an IEI gene panel (**Figure 1A**; **Supplementary Data Sheet 2**). Blood samples for RNA-seq were collected in PAXgene tubes at the first (available) outpatient visit (**Figure 1B**). In addition, each patient was immunophenotyped (total lymphocytes, leukocytes, T cells, B cells, NK cells, and differentiation) at the time of sample collection.

Finally, patient and family data relevant for IEI diagnoses were extracted from the electronic patient file. Since IEI patients may receive therapies that modify the immune system and alter cellular composition, we also extracted which medications had been administered.

### Genomic reanalysis

#### Whole-exome sequencing

To update the annotation of the ES data, we used the called variants from the original VCF files and added the latest annotations using the MOLGENIS Variant Interpretation Pipeline (VIP) version 7.9.1 (19). VIP includes annotations based on the predicted pathogenicity from CAPICE (28), the effect on alternative splicing (SpliceAI) (29), non-coding annotations from GREEN-DB (30), known classifications from the VKGL (31) and Clinvar (32) databases and population frequencies from the genome aggregation database (GnomAD) (33). Using VIP, we also specified a decision tree to prioritize coding and non-coding variants present in the ES data (**Supplementary Data Sheet 3**). Running VIP on all samples on a multicore Linux (Rocky Linux 9.3) computer required at least 1 CPU and 16 GB of RAM with a runtime of 2 hours.

### Transcriptomic analysis

#### Raw data processing

For all 22 patients, RNA was extracted from PAXgene WB samples using the Qiagen Fast Select Kit (Qiagen, GmbH, Hilden, Germany) to remove rRNA and globin mRNA. Next, all samples were prepared and sequenced at GenomeScan (GenomeScan, BV, Leiden, the Netherlands) on the Illumina NovaSeq 6000 platform (Illumina, inc.). During the project, the laboratory standard mRNA NGS library preparation kit used was replaced by GenomeScan. Consequently, 15 samples were prepared using the reverse stranded NEBNext PolyA kit and NEBNext PolyA and NEBNext RNA Ultra II Directional kits (Illumina, Inc.) and 7 samples were prepared with the forward stranded dIDT TruSeq Compatible Plate Duplex RNA kit (Integrated DNA Technologies, Inc, Coralville, IA, USA). To ensure RNA quality, we applied RNA Integrity Number and Transcript Integrity Number threshold values of 7 and 70, respectively. FASTQ files were pre-processed with TrimGalore v0.6.7 (https://www.bioinformatics.babraham.ac.uk/projects/trim_galore/). To maintain consistency with the ES, which were performed using the GRCh37 genome build, RNA−seq reads were aligned to the GRCh37 reference genome. Alignment was carried out with STAR (v2.7.9a) (34) with Gencode (v29) annotations (35), achieving a median read count of 200 million (range 100–600 million reads).

#### RNA-seq outlier analysis

We used a custom implementation (11) of OUTRIDER(v1.20.1) (20), FRASER(v1.99.4)) (21) and the tMAE(v1.0.0) module of DROP (22). In short, OUTRIDER and FRASER identify outlier expression and outlier splicing, which could represent aberrant gene expression and aberrant splicing, for each patient compared to the remainder of the cohort, and optionally a set of background samples. Allele-specific counts for heterozygous single nucleotide variants (SNVs), suggesting mono-allelic expression, are identified by tMAE.

For the detection of outlier expression, featureCounts (R-package v2.16.1) was used to calculate raw transcript counts based on GENCODE (v29, GRCh37). To prevent downstream noise and to improve downstream fitting of the autoencoder model, we removed lowly expressed genes using the default expression filtering procedure implemented in OUTRIDER. Therefore, OUTRIDER retained only genes of which the 95^th^ percentile fragments per kilobase of transcript per million mapped reads (FPKM) exceeded the default cut-off of 1. Next, OUTRIDER calculated size factors as implemented in DESeq2 (36). Using the product of the size factors and estimated counts using the built-in autoencoder, OUTRIDER corrected for technical and biological variation. This was optimized and evaluated by an iterative process that optimizes the area under the precision-recall (auPR) curve of outlier values that have been automatically imputed. Next OUTRIDER modelled these estimated counts for each gene with a negative binomial (NB) distribution, and calculated the *p*-values of the observed counts under this distribution. Multiple testing correction was applied using the Benjamini–Yekutieli method (37) across all transcripts, resulting in adjusted *p*-values. For these values a cut-off of *p-*adjusted < 0.05 was used for statistical significance of outlier expression, as it has been used in different studies that also used OUTRIDER (11, 12, 14). We did not filter the expression outliers based on *z*-scores, a separate standardized value that represents how the observed gene expression deviates from the estimated gene expression.

FRASER counted split and non-split reads from the BAM files and summarized different types of splice junction usage in the Intron Jaccard Index (21). Splice junctions were filtered using FRASER’s internal quality control procedure using a minimum count greater than or equal to 20 in at least one sample. Higher Intron Jaccard Indices represent increased use of canonical splicing, while lower scores indicate a decrease. On the filtered junction counts autoencoder-mediated confounder and noise correction was applied. FRASER estimated *p*−values by modeling the Intron Jaccard Indices for each individual splice junction with a beta-binomial (BB) distribution, and multiple testing correction was applied using the Benjamini–Yekutieli method across all transcripts. Adjusted *p*-values for splice junctions under the fitted BB distribution < 0.05 were considered statistically significant. We chose this cut-off as it has been used in different studies that also used FRASER (11, 16, 21). We did not filter the splicing outliers based on the delta-psi values, which is a separate standardized values that represent how the observed splice junction usage deviates from the estimated splice junction usage.

Mono-allelic expression was assessed using ASEReadCounter (GATK v4.2.4.1) and tMAE, applying a NB test to allele-specific counts at heterozygous SNVs. The change in allele specific expression was summarized in the log2 fold change of the alternative allele ratio. We did not filter the results based on the log2-fold change.

As the authors of DROP have shown that a cohort should contain at least 50–60 samples for OUTRIDER and 30 samples for FRASER for the autoencoder and count estimation (22), we increased the sample size by including an additional set of background samples of 21 unrelated in-house patients. Of these, 8 were prepared using the Qiagen Fast Select and forward stranded dIDT TruSeq Compatible Plate Duplex RNA kits and 13 were prepared using the reverse stranded NEBNext PolyA kit and NEBNext PolyA and NEBNext RNA Ultra II Directional kits. All background samples were sequenced on the NovaSeq 6000 platform. In addition, we included 300 random WB-based external count files from the Genotype-Tissue Expression (GTEx) Project (v6). The quality of correcting for noise and confounding factors by the autoencoder was evaluated. The auPR for OUTRIDER was 0.80 and that for FRASER was 0.69. The heatmaps for OUTRIDER and FRASER showed decreased sample clustering and the PCA plots showed decreased co-variation between the samples after noise and confounder correction for both OUTRIDER and FRASER (**Supplementary Data Sheet 6**). This supports that the current set-up using 43 internally sequenced patients (21 patients and 22 background samples) and 300 external GTEx count files is sufficiently robust for expression and splicing outlier detection.

The complete analysis required ~9 h for our study and background cohorts on a multicore Linux system (Rocky Linux 9.3, ≥8 CPUs, 64 GB RAM).

### Extended gene network search

In seven cases with multiple expression and/or splicing outliers exome-wide, we extended our analysis to detect candidate genes using an unbiased approach. Here we used the GeneNetwork Assisted Diagnostic Optimization (GADO) webtool, which is based on co-expression networks (38, 39). For each case, genes containing expression or splicing outliers from our initial analysis and Human Phenotype Ontology (HPO) terms derived from the patient’s phenotype were submitted to GADO. This resulted in a list of prioritized genes related to the patient’s phenotype. Genes with a *z*-score ≥ 4, indicating significant relevance to the HPO terms, were subsequently evaluated for clinical relevance by our multidisciplinary team through literature review and variant detection in the available ES data.

### Multidisciplinary interpretation

Following the identification of expression outliers and splicing outliers and potentially causal variants, we interpreted the findings in a multidisciplinary team comprising immunologists (rheumatologist A.R., pediatric rheumatologist G.E.L. and pediatric infectiologist R.B.), a medical immunologist (A.J.A.L.), clinical geneticists specialized in IEI (E.Z.H. and C.C.E.T.P.), an immunogenetics researcher (I.J.), clinical genetics laboratory specialists (A.M.H. and M.E.G.) and bioinformatics researchers (W.T.K.M. and T.N.) (**Figure 1C**). Per case, we evaluated if the genes for which expression and splicing outliers were found, matched the clinical phenotype of the patient and contained a reannotated variant. Cases without genes for which expression and splicing outliers were found or without variants in these genes were classified as ‘inconclusive’. When a variant was present, the expected mode of inheritance was determined based on available clinical and familial data. Variants in genes that matched the expected mode of inheritance were categorized as ‘candidate variants’ and evaluated in the context of available literature and expert opinions to identify ‘potentially causal variants’.

## Results

### Patient cohort

We included 22 patients without a molecular diagnosis. Four had received immunomodulatory treatment with rituximab at various time points before the WB sample for the current study was taken. Rituximab is a monoclonal antibody binding to the CD20-antigen, resulting in an induced depletion of B cells. Such a shift in cellular composition will be reflected in the overall gene expression pattern. In our four patients, the influence of rituximab on RNA expression was most pronounced in the patient treated most recently to the WB sample taking (in 2024). In this patient, RNA_PID_020_C, few to no B cells were estimated based on expression-based cell type enrichment analysis and flow cytometry (**Supplementary Data Sheets 4**, **5**). As a result, expression and splicing differences observed in this patient were interpreted in the context of altered cell type composition. Moreover, no consistent pattern in cell type–composition was identified in the other patients.

### Identification of candidate variants

As discussed in the Methods, we performed RNA-seq to assess variation at gene expression splice junction level that could be associated with the clinical phenotype. Per patient, a median of one expression outliers and three splicing outliers for IEI genes were detected. In addition, a median of 48 IEI genes with variants at heterozygous loci showed mono-allelic expression. The significance of all expression and splicing outliers and allele-specific counts at heterozygous SNVs was determined using an adjusted *p-*value < 0.05.

In eight patients, we detected at least one expression or splicing outlier in the RNA-seq with a DNA variant in the same gene that could explain the detected outlier (**Tables 1A, B**). We then evaluated whether the variants segregated with disease. In three cases (RNA_PID_010_C, RNA_PID_014_C, RNA_PID_008_C), the variants were consistent with the expected mode of inheritance and available clinical and familial data (**Table 1A**). Based on the literature, in-depth analysis and expert opinions, we categorized these variants as ‘potentially causal’, and these three cases are discussed in detail below. For five cases, the identified variants were classified as ‘inconclusive’ (**Table 1B**) (**Supplementary Data Sheet 7**). For case RNA_PID_007_C, a genetic variant altering splicing was found in the *PLCG2* gene. Gain-of-function (GOF) effects in *PLCG2* are associated with APLAID (autoinflammation, antibody deficiency, and immune dysregulation) consistent with the patient’s phenotype. Nevertheless, the splice variant identified in this case is expected to result in a loss-of-function (LOF), as RT-PCR and Sanger sequencing of the RNA in our diagnostics department confirmed aberrant splicing with intron retention, leading to the introduction of a premature termination codon (r.1072_1073ins1072 + 1_2072 + 24 p.(Leu358insArgGlu*)), possibly leading to nonsense-mediated decay or a truncated protein. We also observed mono-allelically expressed single nucleotide variants (SNVs) in the *PLCG2* gene for this patient (**Supplementary Data Sheet 7**). Nevertheless, the same variant was also detected in the patient’s unaffected father and in ClinVar, LOF mutations are generally classified as variants of unknown significance.In the other four cases (RNA_PID_004_C, RNA_PID_003_C, RNA_PID_012_C and RNA_PID_022_C), we identified heterozygous likely pathogenic or pathogenic variants in genes associated with autosomal recessive (AR) inheritance that were consistent with the patient’s clinical phenotypes. However, combined transcriptomic analysis, re-analysis of the ES data with VIP and copy number analysis, did not identify a second (likely) pathogenic variant to support this mode of inheritance. For the remaining 14 cases, no aberrant genes in RNA-seq or no DNA-variant in the outlier genes was identified. These cases were therefore also categorized as ‘inconclusive’.

**Table 1A:** Patients with a potential causal variant detected by RNA-sequencing.

Patient ID	Gene	Variant	Effect	Inheritance	Aberrant event	*p*-adjusted	Effect		Total AE genes in IEI panel	Total AS genes in IEI panel	Total MAE genes in IEI panel	Category
RNA_PID_010_C	*IKBKG*	NM_001099857.5: c.671 + 2T>G*	Splice donor variant	XLR	Splicing	FRASER: 7.93e^-7^		Delta-psi: 0.16	1	3	37	Potentially causal variant
RNA_PID_014_C	*CYBB*	NM_000397.4: c.45 + 5G>A*	Splice donor 5^th^ base variant	XLR	Expression, mono-allelic expression	OUTRIDER: 2.87e^-15^ tMAE**: 4.93e^-4^		*z*-score: -9.62 Log2FC: 4.07	1	1	53	Potentially causal variant
RNA_PID_008_C	*NFKB1*	NM_003998.4: c.1495 + 506T>C*	Intron variant	AD	Splicing	FRASER: 0.032		Delta-psi: -0.24		1	43	Potentially causal variant

XLR, X-linked recessive; AD, autosomal dominant; AE, aberrantly expressed; AS, aberrantly spliced; MAE, mono-allelically expressed; *, heterozygous; **, most significant heterozygous SNV detected by tMAE; Log2FC, fold-change of most significant heterozygous SNV detected by tMAE; *z-*score, level of aberrant expression; delta-psi, level of aberrant splicing; Log2FC, level of mono-allelic expression.

**Table 1B:** Patients with a candidate variant from DNA-sequencing evaluated with RNA-sequencing.

Patient ID	Gene	Variant	Effect	Inheritance	Aberrant event	*p*-adjusted	Effect	Total AE genes in IEI panel	Total AS genes in IEI panel	Total MAE genes in IEI panel	Category
RNA_PID_007_C	*PLCG2*	NM_002661.5: c.1072 + 5G>A*	Splice donor 5^th^ base variant	AD	Mono-allelic expression	tMAE**: 8.71e^-6^	Log2FC: -10.37			110	Inconclusive
RNA_PID_004_C	*RIPK1*	NM_001354930.2: c.1844T>C*	Missense variant	AR	Mono-allelic expression	tMAE**: 0.040	Log2FC: -1.11			83	Inconclusive
RNA_PID_003_C	*CARMIL2*	NM_001013838.3: c.787C>T*	Stop gained	AR	Mono-allelic expression	tMAE**: 0.0084	Log2FC: -2.75	1	9	23	Inconclusive
RNA_PID_012_C	*NCKAP1L*	NM_005337.5: c.1625 + 2T>G*	Splice donor variant	AR	Splicing	FRASER: 2.08e^-8^	Delta-psi: -0.16		3	83	Inconclusive
RNA_PID_022_C	*DOCK8*	NM_203447.4: c.1285 + 1G>A*	Splice donor variant	AR	Splicing, mono-allelic expression	FRASER: 0.0014, tMAE**: 4.55e^-4^	Delti-psi: -0.28 Log2FC: 2.01		1	42	Inconclusive

AD, autosomal dominant; AR, autosomal recessive; AE, aberrantly expressed; AS, aberrantly spliced; MAE, mono-allelically expressed; *, heterozygous; **, most significant heterozygous SNV detected by tMAE; Log2FC, fold-change of most significant heterozygous SNV detected by tMAE; *z-*score, level of aberrant expression; delta-psi, level of aberrant splicing; Log2FC, level of mono-allelic expression.

#### Case 1: IKBKG

**Figure 2:**
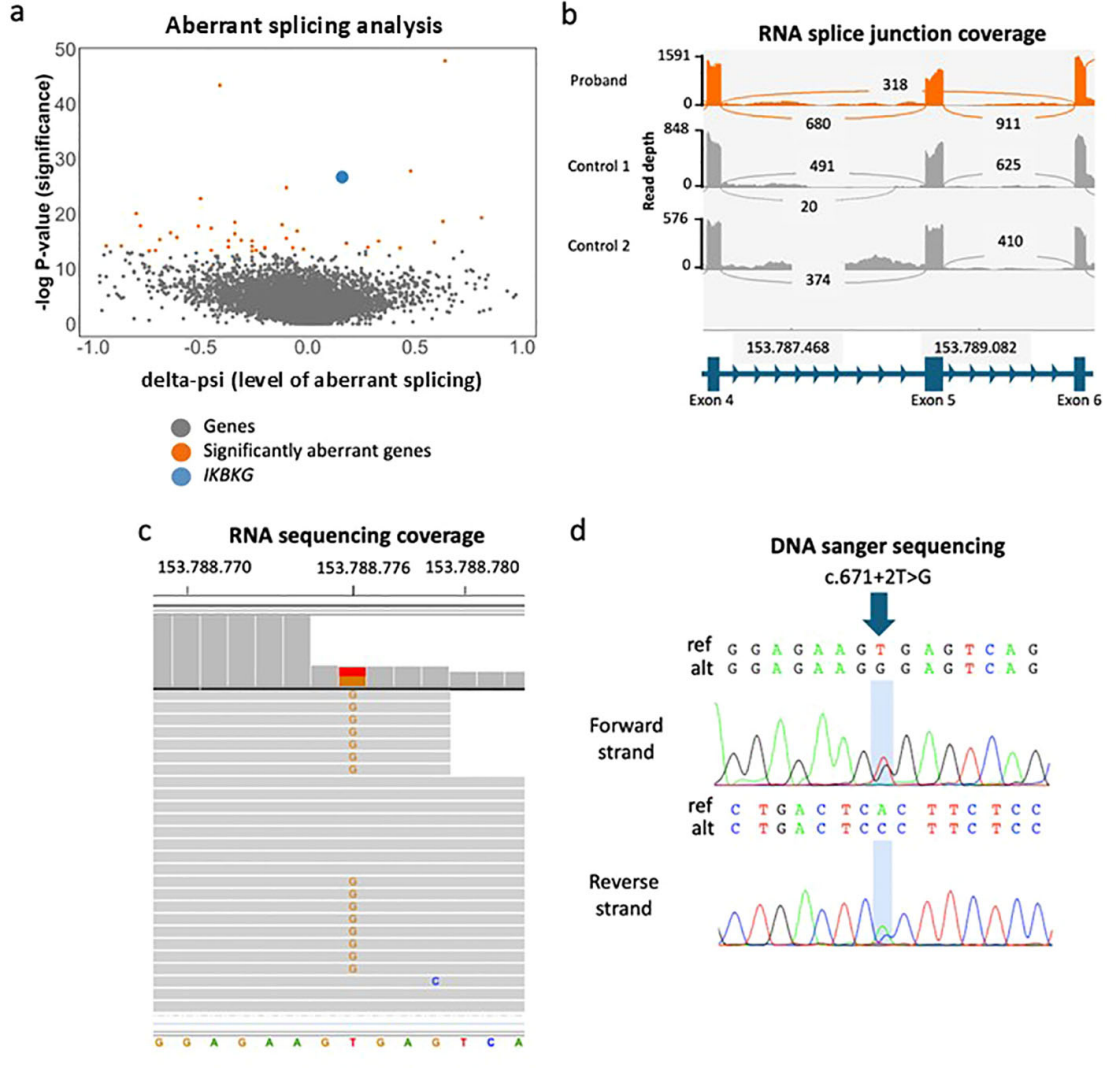
RNA-sequencing detected aberrant splicing in an *IKBKG* case. **(A)** FRASER analysis in a volcano plot. Each dot represents a splicing outlier. Blue dot represents *IKBKG*. Orange dots are genes that are significant (*P*-adjusted < 0.05). X-axis shows a delta logit of the delta-psi value that indicates the level of aberrant splicing. Y-axis shows the *P-*adjusted value representing the significance of the aberrantly spliced genes in the context of all genes of all patients in the complete cohort. **(B)** Sashimi plot showing the RNA-sequencing reads that cover the region around exon 5 of *IKBKG* for the patient and two random controls (no RNA-sequencing data was available for the parents). **(C)** Relative number of reads in RNA-sequencing data for each allele around the variant on genomic position chrX:153,788,776 (c.671 + 2T>G) (GRCh37) that causes skipping of exon 5 in *IKBKG*. **(D)** Sanger sequencing results of the forward and reverse strand of a PCR fragment flanking the variant of interest. The variant at chrX:153,788,776 (c.671 + 2T>G) is indicated by a blue arrow (A>C on reverse strand).

Case 1 reports the 3-month-old son of non-consanguineous parents with a systemic autoinflammatory disease. The patient presented with lobular panniculitis, periodic fever, anemia, thrombocytopenia, hepatosplenomegaly and failure to thrive. Genetic diagnostics with ES, including an IEI gene panel and a single nucleotide polymorphism array, did not detect a (likely) pathogenic variant. RNA-sequencing detected outlier splicing of *IKBKG* transcripts (*P*-adjusted = 7.93e-7)(**Figure 2A**), characterized by skipping of exon 5 skipping in approximately 30% of reads (**Figure 2B**). This splicing defect is likely attributable to an intronic variant in the canonical splice donor site (NM_001099857.5: c.671 + 2T>G) in *IKBKG* at genomic position chrX:153,788,776 (**Figure 2C**). Targeted Sanger sequencing confirmed the variant in the patient (**Figure 2D**) and demonstrated it to be *de novo*, as neither of the unaffected parents carried the variant. Long-range polymerase chain reaction (PCR) revealed a mosaicism, with approximately 40–50% of the patient’s cells carrying the variant. Exon 5 skipping has been reported before, with LOF variants in *IKBKG* resulting in expression of a protein isoform that lacks the domain encoded by exon 5. This disrupts type 1 interferon responses to Toll-Like-Receptor-3 and RIG-1-Like-Receptor stimulation, which normally establish an antiviral host response (40). This mechanism underlies NEMO-deleted exon 5 autoinflammatory syndrome, which manifests with a phenotype similar to that observed in our patient.

#### Case 2: CYBB

**Figure 3:**
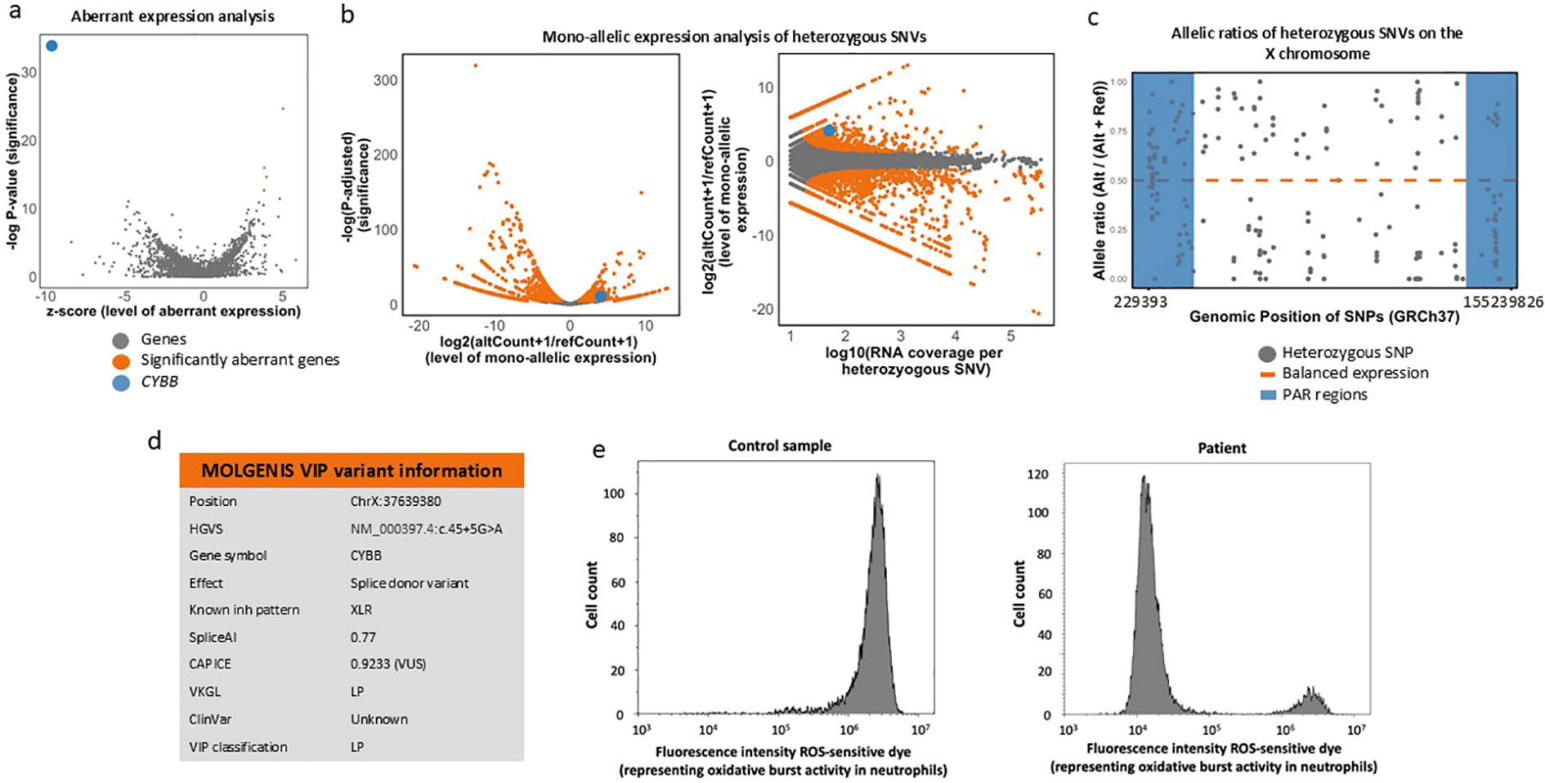
RNA-sequencing detected reduced *CYBB* expression implying skewed X-chromosome inactivation in a carrier of an X-linked recessive disease. **(A)** Results of the OUTRIDER analysis in a volcano plot. Each dot represents an expression outlier. Blue dot represents *CYBB*. Orange dots are genes that are significant (*P*-adjusted < 0.05). X-axis shows a *z*-score that indicates the level of aberrant expression. Y-axis shows the *P-*adjusted value representing the significance of the aberrantly expressed genes in the context of all genes of all patients in the complete cohort. (B1) tMAE analysis. Each dot represents a heterozygous single nucleotide variant (SNV). Blue dots represent heterozygous SNVs in *CYBB*. Orange dots are SNVs that are significant (*P*-adjusted < 0.05). X-axis shows the log2 of the alternative allele ratios that indicates the level of mono-allelic expression. Y-axis shows the *P-*adjusted value representing the significance of the mono-allelically expressed heterozygous SNVs in the context of all heterozygous SNVs of per patients. (B2) tMAE analysis. Each dot represents a heterozygous SNV. Blue dots represent heterozygous SNVs in *CYBB*. X-axis shows the log10 of the RNA-sequencing coverage per heterozygous SNV and the Y-axis shows the log2 of the alternative allele ratios that indicates the level of mono-allelic expression **(C)** Scatter plot showing the proportion of RNA-sequencing reads that cover the different heterozygous SNVs between chrX:6114 and chrX:249,110 (GRCh37). **(D)** Relevant annotations added by VIP to the genetic variant called in WES at chrX:37,639,380 (GRCh37) within *CYBB.* **(E)** After *in vitro* phorbol 12-myristate 13-acetate (PMA) stimulation, an oxidative burst was observed in 10–11% of the patient’s neutrophils, which is reduced compared to a control sample.

Case 2 reports a 20-year-old female presenting with symptoms matching juvenile systemic lupus erythematosus and Crohn’s disease. Her affected father was diagnosed with X-linked recessive chronic granulomatous disease caused by a heterozygous splice variant NM_000397.4: c.45 + 5G>A in *CYBB*. RNA-seq revealed reduced *CYBB* expression in our patient (*p-*adjusted = 2.87e-10)(**Figure 3A**), possibly as the result of nonsense-mediated decay. In addition, skewed allelic expression was detected for a nearby variant at genomic position chrX:37,653,141 (**Figures 3B, C**), potentially resulting in the significantly reduced *CYBB* transcript levels, suggesting skewed X-inactivation. The classical inheritance pattern for *CYBB* mutations is X-linked recessive, but the patient’s clinical phenotype is described in carriers with skewed X-chromosome activation. Consistent with the inheritance pattern, the ES data showed the same heterozygous splice variant in *CYBB*. VIP categorized this variant as likely pathogenic based on the VKGL database (**Figure 3D**). Similar variants have been described to decrease neutrophil function (41–43). Additional functional testing supported the hypothesis of skewed X-chromosome inactivation, with 87-88% of the X-linked AR-gene being methylated and only 10–11% of the neutrophil granulocytes showing a normal oxidative burst after stimulation (**Figure 3E**).

#### Case 3: NFKB1

**Figure 4:**
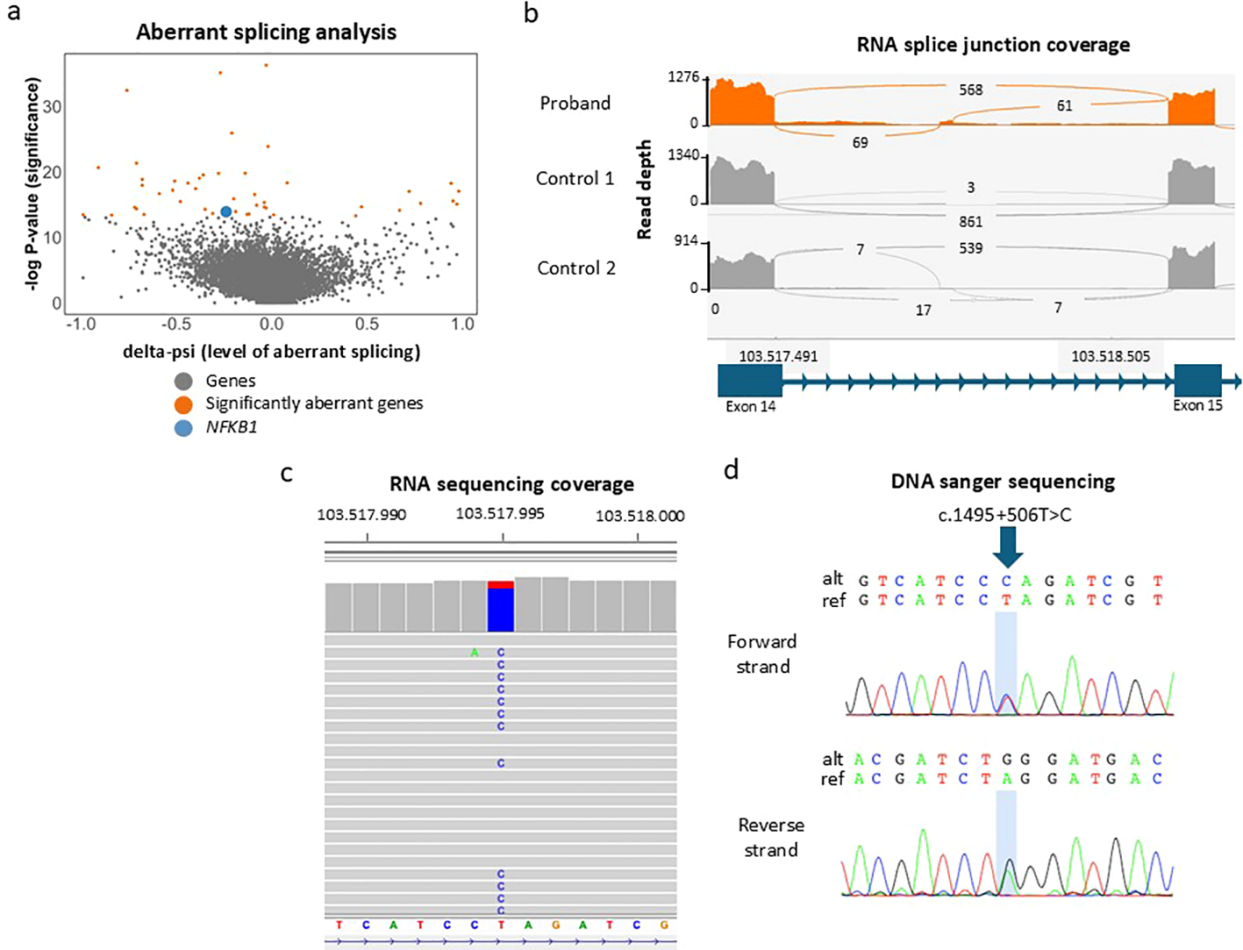
RNA-sequencing detected aberrant splicing of *NFKB1* and a variant in *NFKB1*, indicating additional testing to evaluate its potential role as a causal gene. **(A)** Results of the FRASER analysis in a volcano plot. Each dot represents a splicing outlier. Blue dot represents *NFKB1.* Orange dots are genes that are significant (*P*-adjusted < 0.05). X-axis shows a delta logit of the delta-psi value that indicates the level of aberrant splicing. Y-axis shows the *P-*adjusted value representing the significance of the aberrantly spliced genes in the context of all genes of all patients in the complete cohort. **(B)** Sashimi plot showing the RNA-sequencing reads that cover the region between exons 14 and 15 of *NFKB1* for the patient and two random controls (no RNA-sequencing data was available for the parents). **(C)** Relative number of reads in RNA-sequencing data that cover the variant on position chr4:103,517,995 (GRCh37) that causes the pseudo-exon in *NFKB1*. **(D)** Sanger sequencing results of the forward and reverse strand. The variant at chr4:103,517,995 (c.1495 + 506T>C) is indicated by a black arrow (A>G on reverse strand).

Case 3 reports a female of 42 years with common variable immunodeficiency, lymphadenopathy, immune thrombocytopenic purpura and interstitial lung disease. Her CD21 low B cells are 29.3% and therefore increased (normal is < 10%). The father of the patient’s mother was reported to have died from sepsis after cholecystectomy. The patient’s mother is clinically unaffected. In our patient, transcripts of *NFKB1* were identified to show outlier splicing (*P*-adjusted = 0.03) (**Figure 4A**), resulting in a pseudo-exon between exons 14 and 15 (**Figure 4B**) in 11% of the RNA reads. The inclusion of this exon is a known transcript predicted to result in nonsense-mediated decay due to a premature stop codon, as annotated in the Ensembl database (44). In three other samples, this pseudo-exon occurred in 0%, 0.6% and 1.3% of the RNA reads, confirming an abnormal abundance in the patient. RNA-seq additionally showed a heterozygous deep-intronic variant (NM_003998.4: c.1495 + 506T>C) at genomic position chr4:103,517,995 (GRCh37)(**Figure 4C**), a region not covered (and therefore not detected) by ES. This variant was confirmed with Sanger sequencing (**Figure 4D**), and segregation analysis showed that her mother also carries the variant. Given that possible LOF variants are common pathogenic mechanisms in *NFKB1* and result in phenotypes consistent with our patient, with variable penetrance, we believe this variant is potentially causal for the phenotype (45). For the moment, however, this variant remains classified as a VUS as we need to perform functional validation and RNA-sequencing on whole blood from the patient’s asymptomatic mother, who carries the same variant.

## Discussion

In this study, we show that an RNA-guided approach can complement conventional genetic diagnostics and contribute to the identification of causal variants in IEI patients who did not receive a molecular diagnosis from exome sequencing during diagnostic testing. By integrating aberrant expression analysis, aberrant splicing analysis and mono-allelic expression analysis with multidisciplinary interpretation, we identified potential disease-causing variants for 3 out of 22 IEI cases. We have specifically demonstrated that RNA-seq contributes in four scenarios: interpreting variants by detecting aberrant expression or splicing (Case 1), assessing X-chromosome (in)activation in female carriers of X-linked disorders (Case 2), prioritizing candidate genes when exome sequencing fails to reveal a genetic cause (Case 3) and detecting a second variant when a heterozygous pathogenic variant is identified in recessive disorders (potentially in our inconclusive cases).

In Case 1, routine diagnostics did not reveal a molecular diagnosis, but RNA-seq identified aberrant splicing in *IKBKG* due to an intronic variant at the donor splice site, which led to exon 5 skipping, resulting in a dysfunctional isoform, as earlier described (40). This case shows that RNA-seq data can improve DNA diagnostics in complicated cases involving pseudogenes. In Case 2, RNA-seq detected reduced *CYBB* expression, which implies skewed X-chromosome inactivation, which was the cause of the patient’s phenotype. Without these data, carriership in a female of this X-linked form of CGD would not have lead to a molecular diagnosis. In Case 3, after negative gene panel testing, RNA-seq revealed a heterozygous deep-intronic variant and aberrant splicing of *NFKB1*, generating a pseudo-exon inducing nonsense-mediated decay. This is the result of a predicted premature stop codon. For the moment, however, this variant remains classified as a VUS as we need to perform functional validation and RNA-sequencing on whole blood from the patient’s asymptomatic mother, who carries the same variant.

In total, our approach showed a diagnostic yield of 13.6%. This yield is on par with previous studies employing RNA-guided approaches. Studies on cohorts with diverse Mendelian conditions have reported similar yields, ranging from 4% to 14% (11–13, 16). Notably, a 14% yield was achieved in an IEI cohort by combining GS and RNA-seq, underscoring the potential of leveraging GS data within our RNA-guided approach (16). Increasing the diagnostic yield and defining the genetic diagnosis in IEI is crucial in guiding treatment decisions, including targeted therapies and curative interventions, and for improving clinical outcomes in IEI patients. A previous study demonstrated that defining the molecular diagnosis could confirm hematopoietic stem cell transplantation (HSCT) as a therapeutic option in 42% of cases and identified therapeutic target options for additional 34% of cases (46). Consistent with this, another study in a cohort of 154 patients showed that nearly 80% of 123 patient with a genetic diagnosis have at least one associated clinical management option, including supportive (76.6%), preventive (61.0%) or targeted treatments (40.2%); HSCT or other organ transplantations (3.3%); and gene therapy (5.3%). Importantly, even molecular diagnoses newly identified following reanalysis were frequently actionable, reinforcing the clinical value of comprehensive and iterative genomic testing (47).

Nevertheless, 19 of our cases showed inconclusive results. In four cases, outlier splicing or mono-allelic expression was detected in the recessive genes *RIPK1, CARMIL2, NCKAP1L* or *DOCK8*, but a second variant was not detected, making it impossible to make a molecular diagnosis based on the outlier expression patterns alone. GS could aid in the identification of a second variant in a non-coding region using the current best-in-practice GREEN-DB (30), Regulatory Mendelian Mutation (ReMM) (48), Functional Analysis Through Hidden Markov Models (FATHMM) (49) and non-coding Essential Regulation (ncER) (50) annotations, in combination with the non-coding decision tree of MOLGENIS VIP.

Moreover, in another case, a splice variant in *PLCG2* was predicted to result in a LOF effect, as RT-PCR analysis in our diagnostics department confirmed aberrant splicing with intron retention, leading to the introduction of a premature termination codon (r.1072_1073ins1072 + 1_2072 + 24 p.(Leu358insArgGlu*)), possibly leading to nonsense-mediated decay or a truncated protein (51). We also observed mono-allelically expressed single nucleotide variants (SNVs) in the *PLCG2* gene for this patient (**Supplementary Data Sheet 7**). Nevertheless, the same variant was also detected in the patient’s unaffected father and in ClinVar, LOF mutations are generally classified as variants of unknown significance in Clinvar. We could not identify candidate variants or genes for the remaining cases. For example, a heterozygous pathogenic variant (NM_016038.2:c.258 + 2T>C r.spl)? was detected in the ES data of the *SBDS* gene. No second variant was identified, despite the gene being associated with AR disease. In addition, we were not able to identify the splice variant in the RNA-seq data and there was also no evidence for aberrant splicing or altered expression of *SBDS*. However, transcripts derived from the allele carrying the pathogenic splice variant could undergo nonsense−mediated decay (NMD), hiding a possible splice aberration. Nevertheless, we still see heterozygous SNVs in the raw RNA-seq data. This could be explained by the inhibition of NMD by the PAXgene tubes. Therefore, we decided that we cannot fully confirm that our splice variant is indeed pathogenic. In addition, we included cases for which no monogenic cause of the disease (Chronic Recurrent Multifocal Osteomyelitis in RNA_PID_009_C and sib RNA_PID_009_S) has yet been identified, and current evidence suggests that a multifactorial cause is more likely than a monogenic one (52). Consequently, the probability of detecting a single potential causal gene in these two cases is markedly reduced.

The inconclusive cases might represent the limitations of our study. A technical reason for not identifying candidate variants could be a second variant in non-coding regions, which is beyond the scope of our current ES data. This demonstrates the need for GS in routine genetic diagnostics for IEI and supports the hypothesis of Rozevska et al. (16) that aberrant expression analysis, aberrant splicing analysis and mono-allelic expression analysis could support the interpretation of non-coding and structural variants (16). Another reason could be residual covariation between samples, even though the performance of the normalization and correcting for noise and confounders were on par with literature (11, 21, 27). Possible causes could be biological sources of uncertainty, including the absence or low abundance of specific cell types and, consequently, relevant isoforms and alternatively spliced transcripts that could be involved in the disease (22, 53, 54). **Supplementary Data Sheet 5** shows the results of XCell, and R package to estimate the relative distribution of cell types using the raw gene expression data of the 22 IEI patients. These supplementary results shows the estimations for the relative distribution of the most present cell types. B-cells and CD8+ T-cells are relatively well represented. Nevertheless, we do see that CD4+ T-cells and Eosinophils are slightly less well represented. As far as we know, there is no general database containing immune cell-type specific transcripts. Technologies, such as long-read sequencing and single-cell sequencing will facilitate studying the expression of immune cell type specific transcripts in the future (55).

Another possible cause could be the heterogeneity of the different samples due to use of different RNA library preparation kits or the use of rituximab during treatment of four patients. Of the UMCG samples, including the 22 sample study cohort and 21 sample background cohort, 28 were sequenced using a reverse stranded kit and 15 using a forward stranded kit. In addition, the GTEx RNA data was included to increase statistical power, were produced using an un-stranded kit (https://gtexportal.org/home/methods). Merging cohorts of RNA data processed with different library preparation kits, while increasing the statistical significance of smaller count differences can increase the number of false positive expression and splicing outliers that need to be manually evaluated (22). After normalization and autoencoder-based dimensionality reduction by OUTRIDER and FRASER, the post-correction heatmaps and PCA plots in **Supplementary Data Sheet 6** showed reduced sample clustering and variance along the principal components (**Supplementary Data Sheet 6**). Therefore, we expect that residual structure is sufficiently captured and incorporated into the predicted means modeled by the Negative Binomial (NB) model for OUTRIDER and by the Beta Binomial (BB) model for FRASER (**Supplementary Data Sheet 6**). These results are also supported by similar findings by the authors of the DROP (22) pipeline, who also tested combining a dataset with external counts from GTEx. They concluded that, while this could increase the number of false positives, the sensitivity was still sufficient. Therefore, we expect OUTRIDER and FRASER are able to detect meaningful expression outliers and splicing outliers. These findings suggest that analyses of cohorts incorporating external GTEx counts or other heterogeneous datasets using OUTRIDER and FRASER can still yield biologically meaningful results.

In 7 of the 19 inconclusive cases with multiple expression or splicing outliers, we extended our analysis to detect candidate genes by integrating information in the form of HPO terms and with detected genes showing outlier expression or outlier splicing in GADO. Unfortunately, this unbiased approach did not yield candidate genes that contained potentially causal variants. Although unsuccessful for these cases, the potential of expanding unbiased strategies and incorporating multi-omics data in future analyses remains promising.

In conclusion, our study demonstrates that our RNA-guided approach can aid in the identification of causative molecular variants across four different scenarios and support genetic diagnosis in IEI patients. In addition, we have shown that, while applying our publicly available workflow, comprising of our RNA outlier pipeline and MOLGENIS VIP and integrating in-house generated and publicly available RNA-sequencing data, such as GTEx, we can identify diagnostically relevant outlier expression and splicing. This can support other diagnostic laboratories, while they collect their own homogenized internally sequenced background cohorts to minimize noise and confounding effects in advance. Based on ongoing collaboration within the ERDERA (https://erdera.org/) project, we aim to further improve our workflow to identify non-coding variants in GS data that regulate gene expression as a dominant genetic cause or missed second hit for recessive genes. Finally, we aim to improve effective use of GADO, and sequence-based models that predict variant-specific changes in gene expression, such as Borzoi. This will further support the interpretation of missed variants or VUSs which may have been missed in the undiagnosed cases. Finally, the ongoing implementation of GS and RNA-seq in routine diagnostics, including multiple tissues, will further refine our ability to pinpoint causative genes in a wide spectrum of diseases and accelerate the translation of a genetic diagnosis toward personalized treatment.

## Data Availability

The UMCG raw datasets presented in this article are not readily available because the raw ES and RNA-seq data are protected and cannot be shared publicly under current legal and ethical frameworks. The background cohort of GTEx (v6) samples used for optimizing the correction for covariation was downloaded from Zenodo using https://zenodo.org/records/5638707. Further inquiries can be directed to the corresponding author. The GTEx Project was supported by the Common Fund of the Office of the Director of the National Institutes of Health, and by the National Cancer Institute, National Human Genome Research Institute, National Heart and Lung and Blood Institute, National Institute on Drug Abuse, National Institute of Mental Health, and the National Institute of Neurological Disorders and Stroke. The version of VIP that was used in this study is publicly available on Zenodo at https://doi.org/10.5281/zenodo.15649045. The RNA analysis pipeline is publicly available on Zenodo at https://doi.org/10.5281/zenodo.18740581. Both code repositories are available under the GNU Lesser Public License v3.0. In addition, both repositories are an aggregate work of several tools, each covered by their own license(s), which should also be considered. Finally, we used the web-based application of GADO on https://www.genenetwork.nl/gado (accessed at 27-3-2025).

## Supplementary material

Supplementary Data Sheet 1 Inclusion criteria IEI patients.

Supplementary Data Sheet 2 IEI gene panel.

Supplementary Data Sheet 3 MOLGENIS VIP decision tree.

Supplementary Data Sheet 4 Clinical phenotype data.

Supplementary Data Sheet 5 Cell type distribution.

Supplementary Data Sheet 6 Heatmaps showing performance of correcting for covariation by the autoencoder.

Supplementary Data Sheet 7 Inconclusive cases.
