# Supplementary material for: A multidisciplinary RNA-guided approach to complement genomic analysis of unsolved patients with an inborn error of immunity
Source: Front Immunol. 2026 May 28;17:1829883. doi: 10.3389/fimmu.2026.1829883 (PMC13252776; doi:10.3389/fimmu.2026.1829883)
Supplement: Supplementary Data Sheet 1 — Inclusion criteria IEI patients. [file DataSheet1.docx]

Supplementary data 1. **IEI inclusion criteria**. Inclusion criteria required a strong clinical suspicion of a monogenic IEI despite the absence of a genetic diagnosis through standard diagnostic procedures. Only patients meeting this criterion and lacking a conclusive molecular finding after WES were eligible for inclusion.

Confirmed or probable severe IEI defined by one of the criteria below:

- Cellular/combined immune deficiency
  - Strong suspicion of combined B and T cell defect:
    - CD4+ T cells < 200/µl, CD8+ T cells < 100/µl or < p5 in children according to age-specific normal values
    - Or opportunistic infections
- Humoral immune deficiency
  - Common variable immunodeficiency (CVID) based on ESID (European Society of Immuno-Deficiencies) criteria
  - Strong suspicion of XLA or other severe B cell deficiency
- Auto-immunity
  - Children: 2 or more auto-immune diseases in different organs (blood is one organ)
  - For adults only, inclusion if, in addition to >3 auto-immune diseases, there is lymphopenia, granulocytopenia or hypogammaglobulinemia
- Every IEI that is an indication for stem cell transplantation (HSCT)
- Severe neutropenia
  - Neutrophils < 500/µl and negative testing for anti-granulocyte antibodies
- Defect in granulocyte-function (like oxydative burst, migration, killing)
- Suspicion of syndromic IEI
- Positive family history for IEI
