# Supplementary material for: A multidisciplinary RNA-guided approach to complement genomic analysis of unsolved patients with an inborn error of immunity
Source: Front Immunol. 2026 May 28;17:1829883. doi: 10.3389/fimmu.2026.1829883 (PMC13252776; doi:10.3389/fimmu.2026.1829883)
Supplement: Supplementary Data Sheet 3 — MOLGENIS VIP decision tree. [file DataSheet3.docx]

Supplementary data 3. **The customized decision tree used to filter the output of VIP.** This is the configuration of the decision tree that we used when running MOLGENIS VIP (VIP) in our study. This decision tree can be provided to VIP to reproduce the predicted variant classifications (see https://molgenis.github.io/vip/usage/command-line-options/).

{

"rootNode": "filter",

"nodes": {

"filter": {

"description": "Filter pass",

"type": "BOOL",

"query": {

"field": "FILTER",

"operator": "==",

"value": [

"PASS"

]

},

"outcomeTrue": {

"nextNode": "vkgl"

},

"outcomeFalse": {

"nextNode": "exit_rm"

},

"outcomeMissing": {

"nextNode": "vkgl"

}

},

"vkgl": {

"description": "VKGL classification",

"type": "CATEGORICAL",

"field": "INFO/CSQ/VKGL_CL",

"outcomeMap": {

"P": {

"nextNode": "exit_p"

},

"LP": {

"nextNode": "exit_lp"

},

"VUS": {

"nextNode": "clinVar"

},

"LB": {

"nextNode": "exit_lb"

},

"B": {

"nextNode": "exit_b"

}

},

"outcomeMissing": {

"nextNode": "clinVar"

},

"outcomeDefault": {

"nextNode": "clinVar"

}

},

"clinVar": {

"description": "ClinVar classification",

"type": "BOOL_MULTI",

"fields": [

"INFO/CSQ/clinVar_CLNSIG"

],

"outcomes": [

{

"description": "Conflict",

"queries": [

{

"field": "INFO/CSQ/clinVar_CLNSIG",

"operator": "contains_any",

"value": [ "Conflicting_interpretations_of_pathogenicity" ]

}

],

"outcomeTrue": {

"nextNode": "chrom"

}

},

{

"description": "LP/P",

"queries": [

{

"field": "INFO/CSQ/clinVar_CLNSIG",

"operator": "contains_any",

"value": [ "Likely_pathogenic", "Pathogenic" ]

}

],

"outcomeTrue": {

"nextNode": "exit_lp"

}

},

{

"description": "VUS",

"queries": [

{

"field": "INFO/CSQ/clinVar_CLNSIG",

"operator": "contains_any",

"value": [ "Uncertain_significance" ]

}

],

"outcomeTrue": {

"nextNode": "chrom"

}

},

{

"description": "B/LB",

"queries": [

{

"field": "INFO/CSQ/clinVar_CLNSIG",

"operator": "contains_any",

"value": [ "Likely_benign", "Benign" ]

}

],

"outcomeTrue": {

"nextNode": "exit_lb"

}

}

],

"outcomeDefault": {

"nextNode": "chrom"

},

"outcomeMissing": {

"nextNode": "chrom"

}

},

"chrom": {

"description": "Chromosome 1-22-X-Y-MT",

"type": "BOOL",

"query": {

"field": "#CHROM",

"operator": "in",

"value": [

"chr1",

"chr2",

"chr3",

"chr4",

"chr5",

"chr6",

"chr7",

"chr8",

"chr9",

"chr10",

"chr11",

"chr12",

"chr13",

"chr14",

"chr15",

"chr16",

"chr17",

"chr18",

"chr19",

"chr20",

"chr21",

"chr22",

"chrX",

"chrY",

"chrM"

]

},

"outcomeTrue": {

"nextNode": "gnomAD"

},

"outcomeFalse": {

"nextNode": "exit_rm"

},

"outcomeMissing": {

"nextNode": "gnomAD"

}

},

"gnomAD": {

"description": "gnomAD QC filter failure",

"type": "EXISTS",

"field": "INFO/CSQ/gnomAD_QC",

"outcomeTrue": {

"nextNode": "gene"

},

"outcomeFalse": {

"nextNode": "gnomAD_AF"

}

},

"gnomAD_AF": {

"description": "gnomAD",

"type": "BOOL_MULTI",

"fields": [

"INFO/CSQ/gnomAD_FAF99",

"INFO/CSQ/gnomAD_HN"

],

"outcomes": [

{

"description": "Filtering allele Frequency (99% confidence) >= 0.02 or Number of Homozygotes > 5",

"operator": "OR",

"queries": [

{

"field": "INFO/CSQ/gnomAD_FAF99",

"operator": ">=",

"value": 0.02

},

{

"field": "INFO/CSQ/gnomAD_HN",

"operator": ">=",

"value": 5

}

],

"outcomeTrue": {

"nextNode": "exit_lb"

}

}

],

"outcomeDefault": {

"nextNode": "gene"

},

"outcomeMissing": {

"nextNode": "gene"

}

},

"gene": {

"description": "Gene exists",

"type": "EXISTS",

"field": "INFO/CSQ/Gene",

"outcomeTrue": {

"nextNode": "sv"

},

"outcomeFalse": {

"nextNode": "greendb_constraint_pro"

}

},

"sv": {

"description": "Structural Variant?",

"type": "EXISTS",

"field": "INFO/SVTYPE",

"outcomeTrue": {

"nextNode": "str"

},

"outcomeFalse": {

"nextNode": "spliceAI"

}

},

"str": {

"description": "Short tandem repeat?",

"type": "BOOL",

"query": {

"field": "INFO/SVTYPE",

"operator": "==",

"value": "STR"

},

"outcomeTrue": {

"nextNode": "str_status"

},

"outcomeFalse": {

"nextNode": "annotSV"

},

"outcomeMissing": {

"nextNode": "annotSV"

}

},

"str_status": {

"description": "Stranger str status (normal, pre_mutation, mutation)",

"type": "CATEGORICAL",

"field": "INFO/STR_STATUS",

"outcomeMap": {

"full_mutation": {

"nextNode": "exit_lp"

},

"pre_mutation": {

"nextNode": "exit_vus"

},

"normal": {

"nextNode": "exit_lb"

}

},

"outcomeMissing": {

"nextNode": "exit_vus"

},

"outcomeDefault": {

"nextNode": "exit_vus"

}

},

"annotSV": {

"description": "AnnotSV classification",

"type": "CATEGORICAL",

"field": "INFO/CSQ/ASV_ACMG_class",

"outcomeMap": {

"5": {

"nextNode": "exit_p"

},

"4": {

"nextNode": "exit_lp"

},

"3": {

"nextNode": "exit_vus"

},

"2": {

"nextNode": "exit_lb"

},

"1": {

"nextNode": "exit_b"

}

},

"outcomeMissing": {

"nextNode": "spliceAI"

},

"outcomeDefault": {

"nextNode": "spliceAI"

}

},

"spliceAI": {

"description": "SpliceAI prediction",

"type": "BOOL_MULTI",

"fields": [

"INFO/CSQ/SpliceAI_pred_DS_AG",

"INFO/CSQ/SpliceAI_pred_DS_AL",

"INFO/CSQ/SpliceAI_pred_DS_DG",

"INFO/CSQ/SpliceAI_pred_DS_DL"

],

"outcomes": [

{

"description": "Delta score (acceptor/donor gain/loss) > 0.42",

"operator": "OR",

"queries": [

{

"field": "INFO/CSQ/SpliceAI_pred_DS_AG",

"operator": ">",

"value": 0.42

},

{

"field": "INFO/CSQ/SpliceAI_pred_DS_AL",

"operator": ">",

"value": 0.42

},

{

"field": "INFO/CSQ/SpliceAI_pred_DS_DG",

"operator": ">",

"value": 0.42

},

{

"field": "INFO/CSQ/SpliceAI_pred_DS_DL",

"operator": ">",

"value": 0.42

}

],

"outcomeTrue": {

"nextNode": "exit_lp"

}

},

{

"description": "Delta score (acceptor/donor gain/loss) > 0.13",

"operator": "OR",

"queries": [

{

"field": "INFO/CSQ/SpliceAI_pred_DS_AG",

"operator": ">",

"value": 0.13

},

{

"field": "INFO/CSQ/SpliceAI_pred_DS_AL",

"operator": ">",

"value": 0.13

},

{

"field": "INFO/CSQ/SpliceAI_pred_DS_DG",

"operator": ">",

"value": 0.13

},

{

"field": "INFO/CSQ/SpliceAI_pred_DS_DL",

"operator": ">",

"value": 0.13

}

],

"outcomeTrue": {

"nextNode": "exit_vus"

}

}

],

"outcomeDefault": {

"nextNode": "utr5"

},

"outcomeMissing": {

"nextNode": "utr5"

}

},

"greendb_constraint_pro": {

"description": "GREEN-DB constraint score promoter",

"type": "BOOL",

"query": {

"field": "INFO/CSQ/GDB_PRO",

"operator": ">",

"value": 0.7

},

"outcomeTrue": {

"nextNode": "nc_general_predictors_ncer"

},

"outcomeFalse": {

"nextNode": "greendb_constraint_enh"

},

"outcomeMissing": {

"nextNode": "greendb_constraint_enh"

}

},

"greendb_constraint_enh": {

"description": "GREEN-DB constraint score enhancer",

"type": "BOOL",

"query": {

"field": "INFO/CSQ/GDB_ENH",

"operator": ">",

"value": 0.7

},

"outcomeTrue": {

"nextNode": "nc_general_predictors_ncer"

},

"outcomeFalse": {

"nextNode": "greendb_constraint_biv"

},

"outcomeMissing": {

"nextNode": "greendb_constraint_biv"

}

},

"greendb_constraint_biv": {

"description": "GREEN-DB constraint score bivalent",

"type": "BOOL",

"query": {

"field": "INFO/CSQ/GDB_BIV",

"operator": ">",

"value": 0.7

},

"outcomeTrue": {

"nextNode": "nc_general_predictors_ncer"

},

"outcomeFalse": {

"nextNode": "greendb_constraint_sil"

},

"outcomeMissing": {

"nextNode": "greendb_constraint_sil"

}

},

"greendb_constraint_sil": {

"description": "GREEN-DB constraint score silencer",

"type": "BOOL",

"query": {

"field": "INFO/CSQ/GDB_SIL",

"operator": ">",

"value": 0.7

},

"outcomeTrue": {

"nextNode": "nc_general_predictors_ncer"

},

"outcomeFalse": {

"nextNode": "greendb_constraint_ins"

},

"outcomeMissing": {

"nextNode": "greendb_constraint_ins"

}

},

"greendb_constraint_ins": {

"description": "GREEN-DB constraint score insulator",

"type": "BOOL",

"query": {

"field": "INFO/CSQ/GDB_INS",

"operator": ">",

"value": 0.7

},

"outcomeTrue": {

"nextNode": "nc_general_predictors_ncer"

},

"outcomeFalse": {

"nextNode": "exit_rm"

},

"outcomeMissing": {

"nextNode": "exit_rm"

}

},

"nc_general_predictors_ncer": {

"description": "ncer non-coding scores",

"type": "BOOL_MULTI",

"fields": [

"INFO/CSQ/ncER"

], "outcomes": [

{

"description": "Any of the general non-coding predictors above green-varan threshold",

"queries": [

{

"field": "INFO/CSQ/ncER",

"operator": ">",

"value": 98.6

}

],

"outcomeTrue": {

"nextNode": "exit_vus"

}

}

],

"outcomeDefault": {

"nextNode": "nc_general_predictors_fathmmmkl"

},

"outcomeMissing": {

"nextNode": "nc_general_predictors_fathmmmkl"

}

},

"nc_general_predictors_fathmmmkl": {

"description": "FATHMM_MKL non-coding scores",

"type": "BOOL_MULTI",

"fields": [

"INFO/CSQ/FATHMM_MKL_NC"

], "outcomes": [

{

"description": "Any of the general non-coding predictors above green-varan threshold",

"queries": [

{

"field": "INFO/CSQ/FATHMM_MKL_NC",

"operator": ">",

"value": 0.908

}

],

"outcomeTrue": {

"nextNode": "exit_vus"

}

}

],

"outcomeDefault": {

"nextNode": "nc_general_predictors_remm"

},

"outcomeMissing": {

"nextNode": "nc_general_predictors_remm"

}

},

"nc_general_predictors_remm": {

"description": "ReMM non-coding scores",

"type": "BOOL_MULTI",

"fields": [

"INFO/CSQ/ReMM"

], "outcomes": [

{

"description": "Any of the general non-coding predictors above green-varan threshold",

"queries": [

{

"field": "INFO/CSQ/ReMM",

"operator": ">",

"value": 0.963

}

],

"outcomeTrue": {

"nextNode": "exit_vus"

}

}

],

"outcomeDefault": {

"nextNode": "gene_back_to_coding_classification"

},

"outcomeMissing": {

"nextNode": "gene_back_to_coding_classification"

}

},

"gene_back_to_coding_classification": {

"description": "Gene exists",

"type": "EXISTS",

"field": "INFO/CSQ/Gene",

"outcomeTrue": {

"nextNode": "exit_lb"

},

"outcomeFalse": {

"nextNode": "exit_rm"

}

},

"utr5": {

"description": "5' UTR",

"type": "EXISTS",

"field": "INFO/CSQ/five_prime_UTR_variant_consequence",

"outcomeTrue": {

"nextNode": "exit_vus"

},

"outcomeFalse": {

"nextNode": "capice"

}

},

"capice": {

"description": "CAPICE prediction > 0.5",

"type": "BOOL",

"query": {

"field": "INFO/CSQ/CAPICE_SC",

"operator": ">",

"value": 0.5

},

"outcomeTrue": {

"nextNode": "exit_lp"

},

"outcomeFalse": {

"nextNode": "greendb_constraint_pro"

},

"outcomeMissing": {

"nextNode": "exit_vus"

}

},

"exit_rm": {

"description": "Remove",

"type": "LEAF",

"class": "LQ"

},

"exit_b": {

"description": "Benign",

"type": "LEAF",

"class": "B"

},

"exit_lb": {

"description": "Likely Benign",

"type": "LEAF",

"class": "LB"

},

"exit_vus": {

"description": "Uncertain Significance",

"type": "LEAF",

"class": "VUS"

},

"exit_lp": {

"description": "Likely Pathogenic",

"type": "LEAF",

"class": "LP"

},

"exit_p": {

"description": "Pathogenic",

"type": "LEAF",

"class": "P"

}

}

}
