# Supplementary material for: A multidisciplinary RNA-guided approach to complement genomic analysis of unsolved patients with an inborn error of immunity
Source: Front Immunol. 2026 May 28;17:1829883. doi: 10.3389/fimmu.2026.1829883 (PMC13252776; doi:10.3389/fimmu.2026.1829883)
Supplement: Supplementary Data Sheet 4 — Clinical phenotype data. [file DataSheet4.docx]

Supplementary data 4. **Clinical and phenotype data of inconclusive cases.**

|  | **Patient ID** | **Phenotype** | **Candidate gene** |
| --- | --- | --- | --- |
| 1 | RNA_PID_007_C | Periodic fever, headache, abdominal pain, elevated CRP, anti-IL1 is effective | *PLCG2* |
|  | RNA_PID_007_P | Unaffected parent of RNA_PID_007_C | - |
| 2 | RNA_PID_004_C | Status after meningitis (haemophilus influenza b), low lymphocytes and gamma globulins. Speech and language developmental delay | *RIPK1* |
| 3 | RNA_PID_003_C | Recurrent hemophagocytosis with fever, skin involvement, diffuse lymphadenopathy, myositis. Potential consanguine parents. Rituximab treatment in 2011. | *CARMIL2* |
| 4 | RNA_PID_012_C | Chronic inflammatory disorder, including periodic fever, fatigue, myalgia, aphthae, chronic elevated CRP/BSE | *NCKAP1* |
| 5 | RNA_PID_022_C | Lymphopenia (CD4⁺ and CD8⁺ T cells), normal immunoglobulins, auto-immunity (antiphospholipid syndrome, vitiligo), ulcerative colitis, anemia, hepatomegaly, fatigue.  WES: DOCK8 c.1285+1G>A, heterozygous, VUS | *DOCK8* |
| 6 | RNA_PID_005_C | Chronic rhinosinusitis, hidradenitis, intertrigo and cheilitis angularis. Single event with fever and elevated CRP, presumably a bacterial infection. Auto-immunity: psoriasis, alopecia areata, auto-immune cytopenia. Lymphoproliferation: T cell Large Granular lymphocyte. Low B cells, normal B cell subsets. Insufficient antibody response after vaccination with tetanus, prenevar and pneumovax. Hypogammaglobulinemia (low IgA and IgM) | *CARD11* |
| 7 | RNA_PID_006_C | Hypogammaglobulinemia, hemophagocytic syndrome after EVB, absent B cells, colitis. Rituximab treatment in 2016. | - |
| 8 | RNA_PID_009_C | Chronic Recurrent Multifocal Osteomyelitis, tonsil hypertrophy |  |
| 9 | RNA_PID_009_S | Sib of RNA_PID_009_C  Chronic Recurrent Multifocal Osteomyelitis, dermatitis, Henoch-Schönlein purpura with dysmorphic erythrocytes. |  |
| 10 | RNA_PID_011_C | Clinical severe Familial Mediterranean Fever in remission with colchicine and canakinumab and heterozygous variant in *MVK* |  |
| 11 | RNA_PID_013_C | Hypogammaglobulinemia, asthma and arthritis | - |
| 12 | RNA_PID_017_C | Developmental delay, immune dysregulation (episodic fever, infections), variable neurological deficits (including right facial paralysis, myalgia), tone dysregulation (hypertonia, pes planea), notable frontal arachnoid calcifications (possibly after meningitis) and ventriculomegaly, epilepsy onset at age 1 year, variable hepatosplenomegaly, chronic microcytic anemia, four finger line on both hands, small little fingernails. Consanguineous parents. | - |
| 13 | RNA_PID_020_C | ANCA-associated vasculitis (type GPA/PR3-ANCA) age of onset 65 years. Rituximab treatment in 2024. |  |
| 14 | RNA_PID_018_C | Clinical diagnosis Muckle-Wells syndrome: periodic fever with elevated infection parameters, headache, erythema, elevated serum amyloid A | - |
| 15 | RNA_PID_021_C | Clinical diagnosis is hyper IgE syndrome: recurrent infections (lung, ENT, skin) onset in childhood, asthma, eczema, oligodontia, repeated bone fractures. Mild bronchiectasis, high IgE (>1300 kU/L) and a VUS in *IL6ST* (c.193A>G p.Ile65Val).  Family ‒ Daughter: bone fractures, oligodontia, viral infections (possibly affected). Father: childhood skin infections. | *IL6ST* |
| 16 | RNA_PID_023_C | Recurrent infections (respiratory tract, urinary tract, ENT, conjunctivitis), asthma, pollen allergy, strongly elevated IgE and eosinophilia.  Family ‒ Brother: colitis, skin infections. Mother: died at 55 from multi-organ failure due to refractory shock in the context of decompensated liver cirrhosis caused by small-duct primary sclerosing cholangitis; no eosinophilia; cardiology report showed no TAAD | *RSPH9* |
| 17 | RNA_PID_016_C | Eosinophilia, eosinophilic esophagitis, recurrent infections (ENT), asthma, eczema, erythema nodosum. Raynaud, mild hypogammaglobulinemia, arthritis.  Brother (deceased) with similar phenotype | - |
| 18 | RNA_PID_002_C | Neutropenia at onset of 23 years of age. Fatigue, myalgia, abdominal infection eci. Recurrent infections (ENT) and aphthae in childhood.  WES: SBDS Chr7(GRCh37):g.66459197A>G NM_016038.2:c.258+2T>C r.spl?; heterozygous, pathogenic | - |
| 19 | RNA_PID_015_C | Elevated erythrocyte sedimentation rate (between 33 and 62 mm/h (normally < 20mm/h), measured between 2018 and 2023) | Missense VUS in *TNFRAIP3* |
