# Supplementary material for: A multidisciplinary RNA-guided approach to complement genomic analysis of unsolved patients with an inborn error of immunity
Source: Front Immunol. 2026 May 28;17:1829883. doi: 10.3389/fimmu.2026.1829883 (PMC13252776; doi:10.3389/fimmu.2026.1829883)

Supplementary data 5. **Cell type distributions.** The XCell R package (v1.1.0) was used to calculate the relative distribution cell types using the raw gene expression data of the 22 IEI patients. The enrichment scores for a subset comprised of CD4+ T cells, CD8+ T cells, B cells, and Eosinophils is shown.


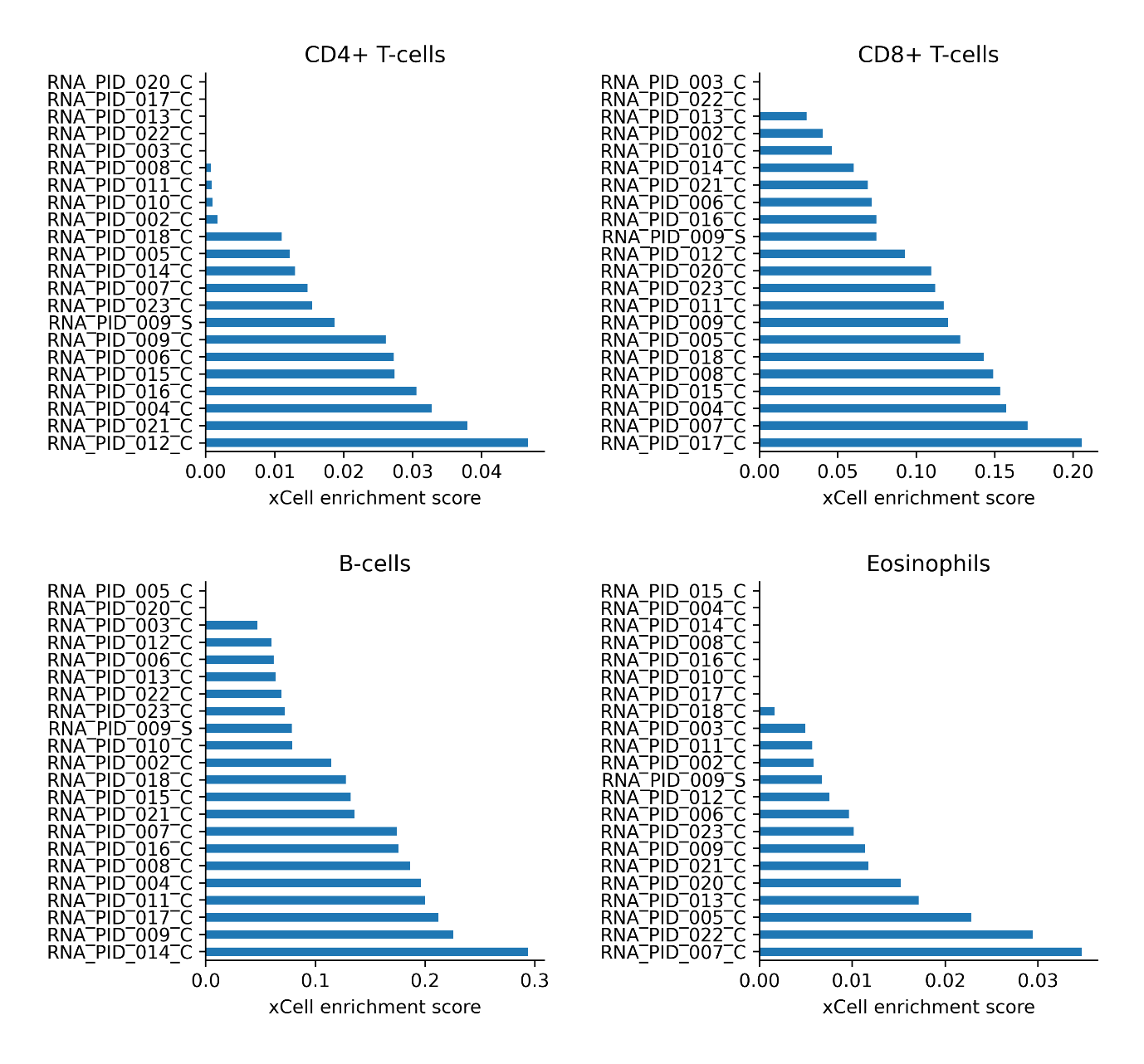

Supplement: Supplementary Data Sheet 5 — Cell type distribution. [file DataSheet5.docx]
