# Supplementary material for: A multidisciplinary RNA-guided approach to complement genomic analysis of unsolved patients with an inborn error of immunity
Source: Front Immunol. 2026 May 28;17:1829883. doi: 10.3389/fimmu.2026.1829883 (PMC13252776; doi:10.3389/fimmu.2026.1829883)
Supplement: Supplementary Data Sheet 6 — Heatmaps showing performance of correcting for covariation by the autoencoder. [file DataSheet6.docx]

## Supplementary data 6: Heatmaps and PCA plots showing performance of correcting for noise and covariation by the autoencoder


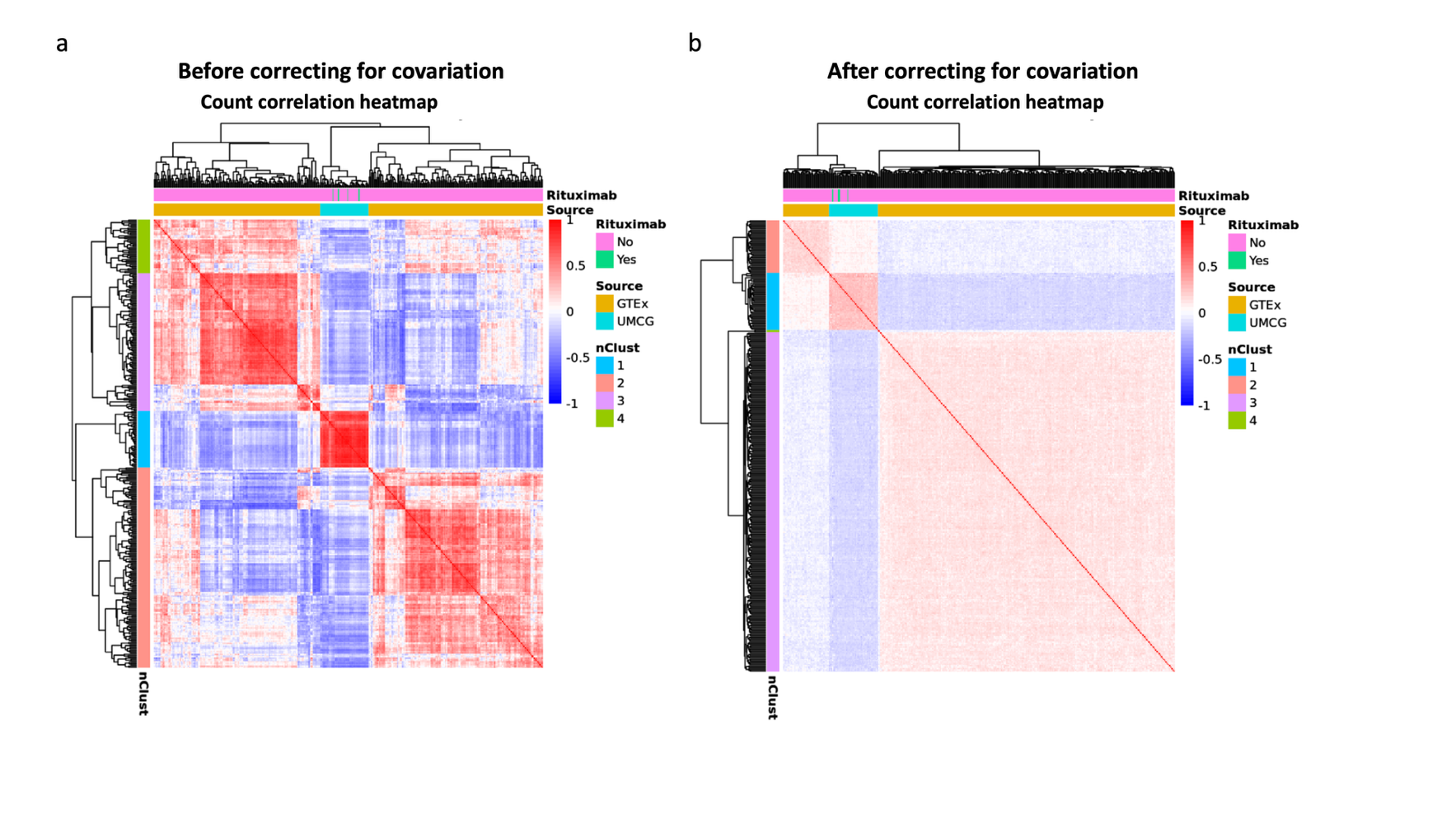

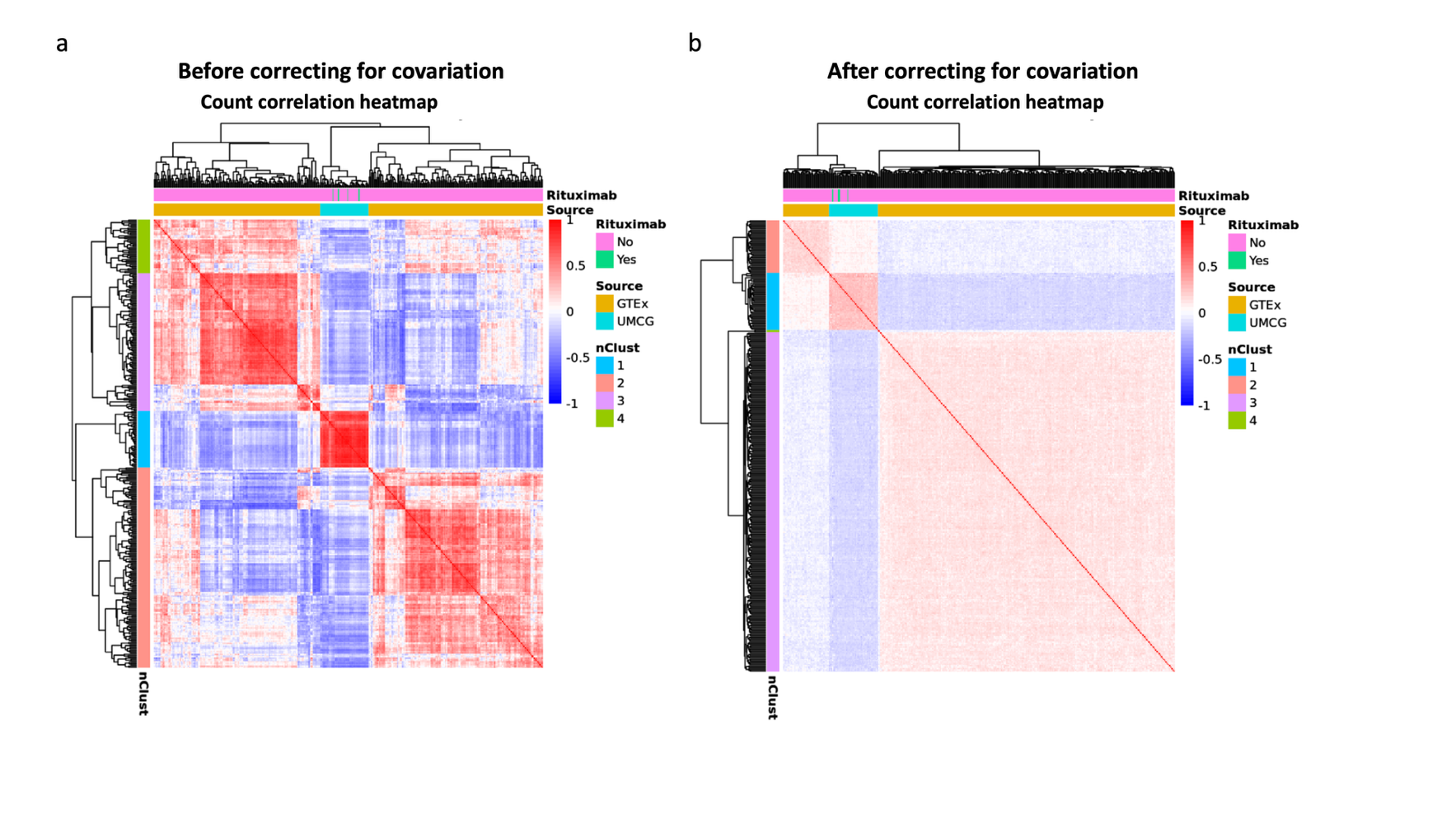

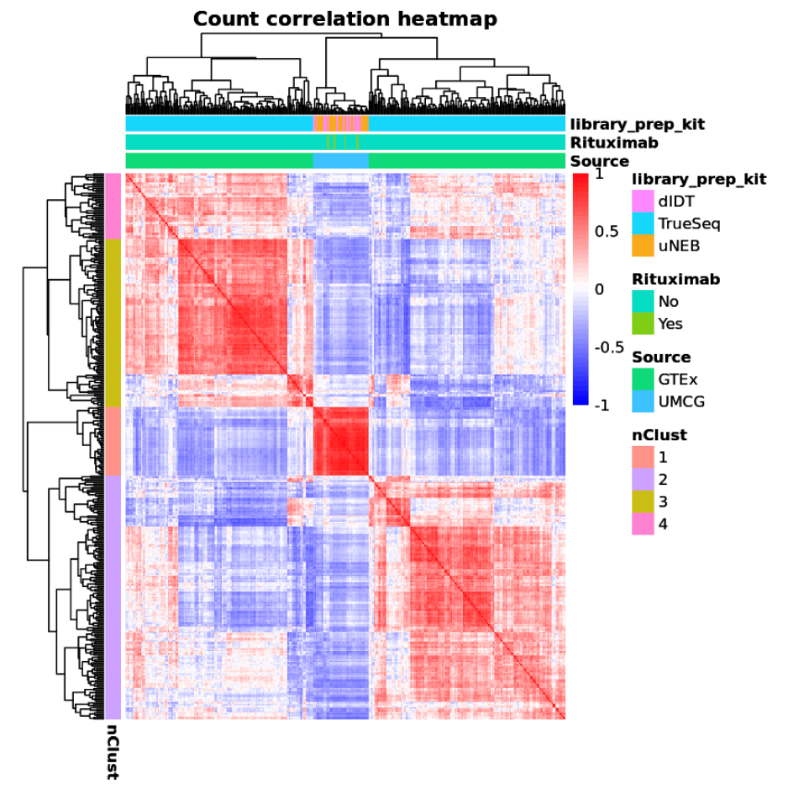

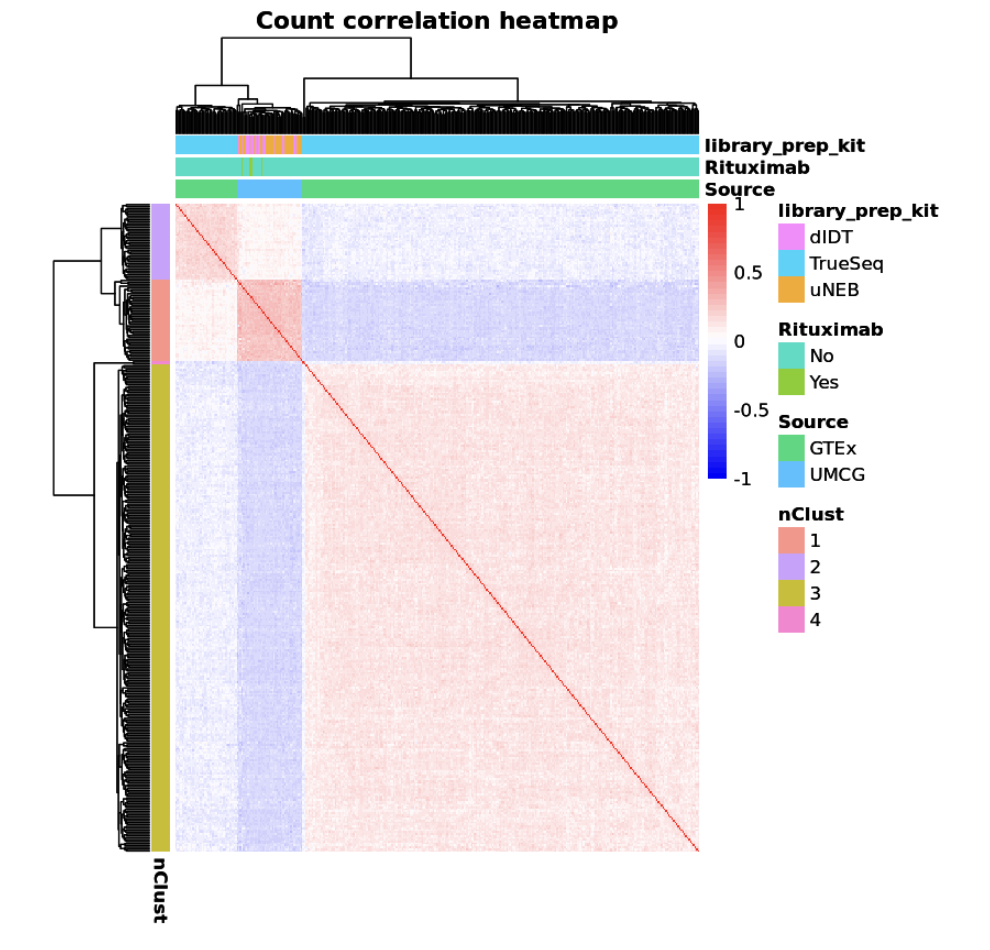


**OUTRIDER noise and confounder correction results**

These heatmaps show the correlation between read counts of the different whole blood samples. **a**, Shows the correlation before noise and confounder correction by the autoencoder. **b**, Shows the correlation after noise and confounder correction by the autoencoder. For this dataset, the optimal amount of encoding dimensions determined by the autoencoder is 50 with an area under the precision-recall (auPR) curve of 0.80. The correlation values can range from -1 (blue) to 1 (red), and clustering of samples is represented by the clustering trees and colours in the top row of the figure. In addition, the samples are annotated with whether they are collected at the UMCG and from GTEx, with whether they received rituximab treatment and which library preparation kit was used. The heatmap after correcting for covariation shows slightly more residual covariation than Oquendo *et al*. and less covariation than Maassen *et al.* (1,2).


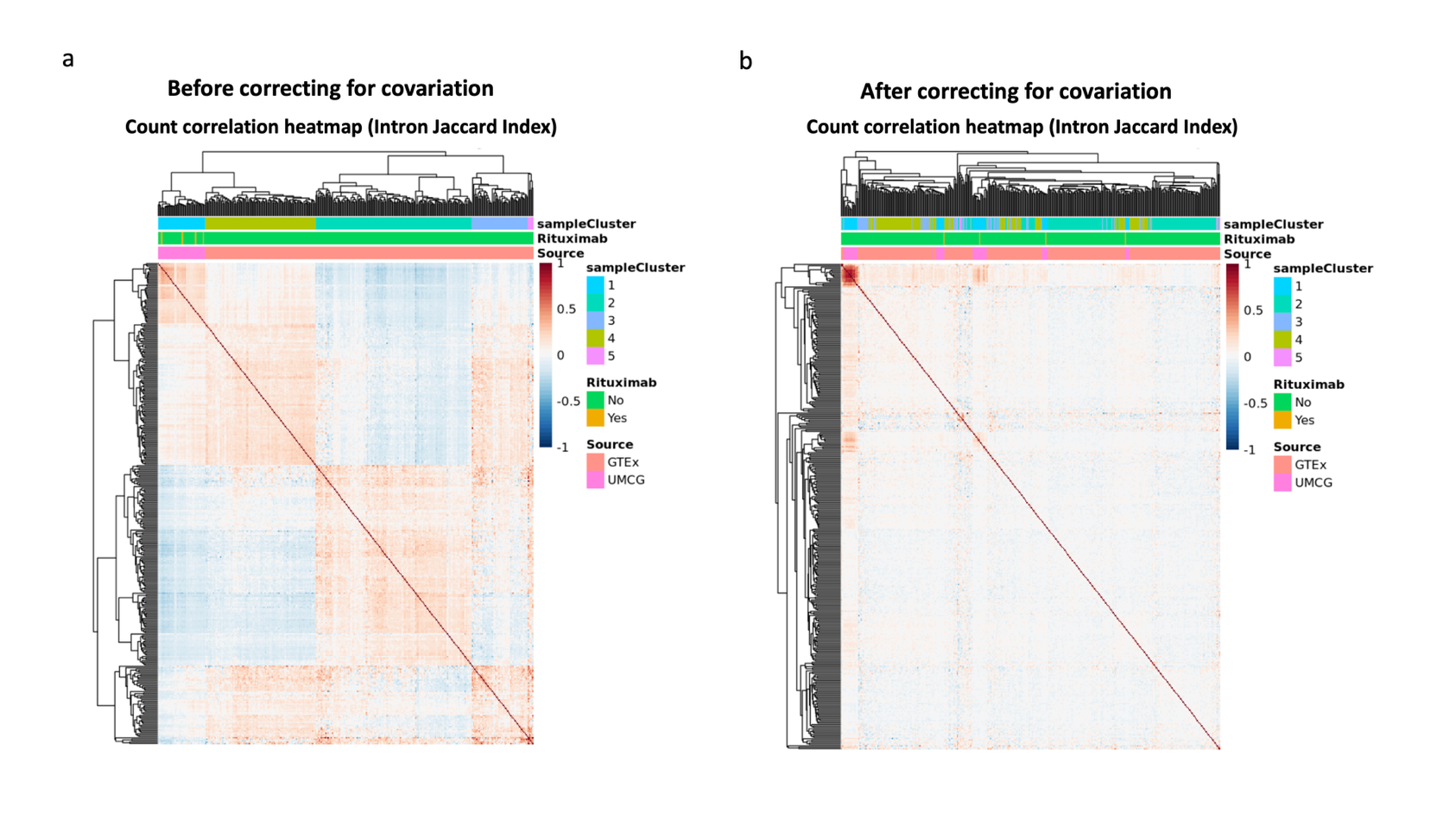


**
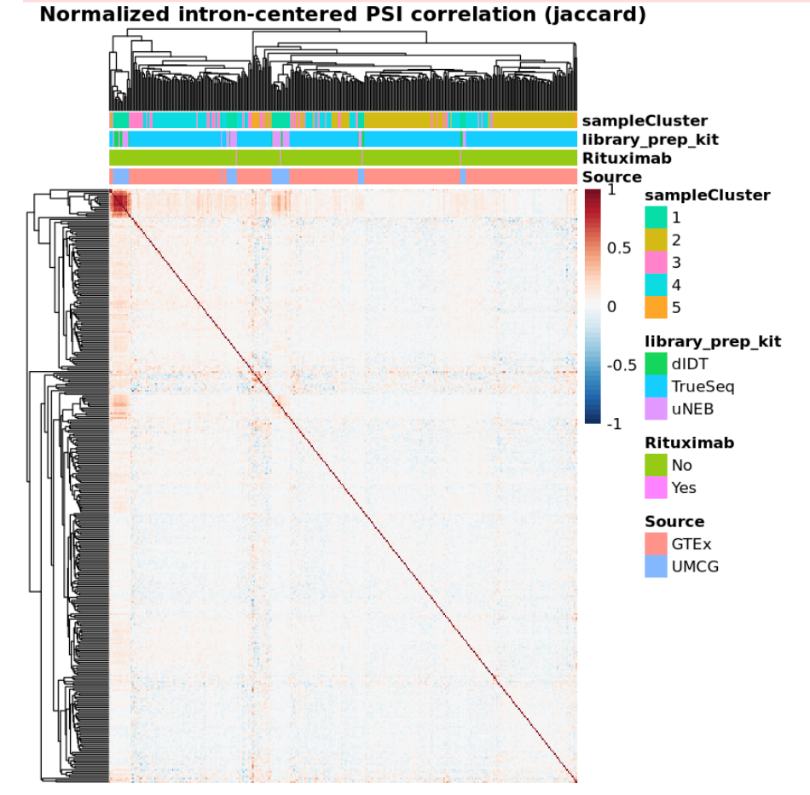
**
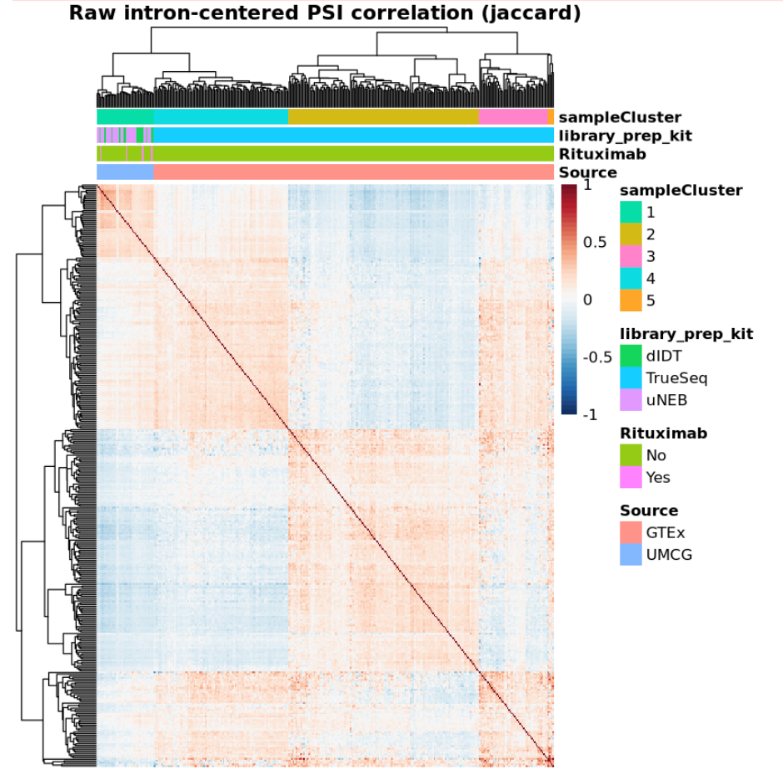


**FRASER noise and confounder correction results**

These heatmaps show the correlation between the Intron Jaccard Index splice metric of the different whole blood samples. This metric represents the usage of specific donor and acceptor splice sites based on split and non-split reads. **a**, Shows the correlation before noise and confounder correction by the autoencoder. **b**, Shows the correlation after noise and confounder correction by the autoencoder. For this dataset, the optimal amount of encoding dimensions determined by the autoencoder is 27 with an area under the auPR curve of 0.69. Correlation values can range from -1 (blue) to 1 (red), and clustering of samples is represented by the clustering trees and colours in the top row of the figure. In addition, the samples are annotated with whether they are samples collected at the UMCG and from GTEx, with whether they received rituximab treatment and which library preparation kit was used. The auPR value for splicing effects is slightly lower compared to Scheller *et al.* and Maassen *et al*.(2,3).


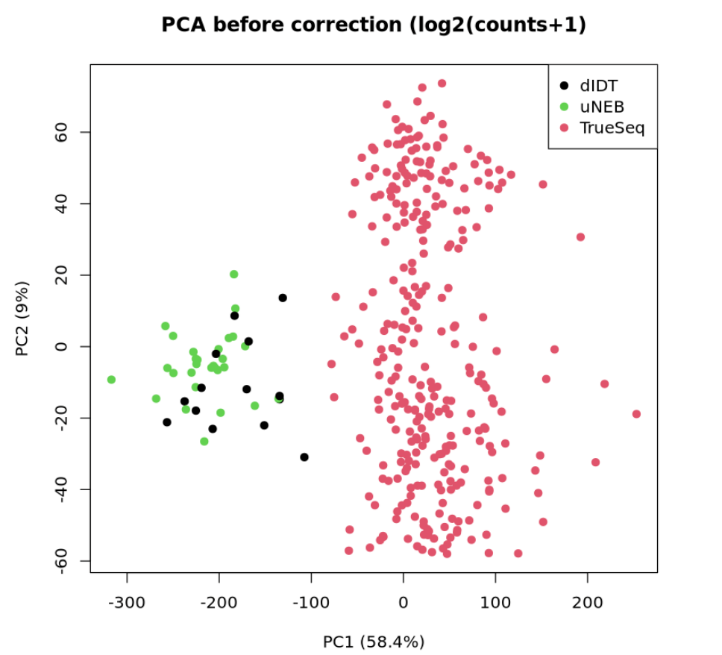

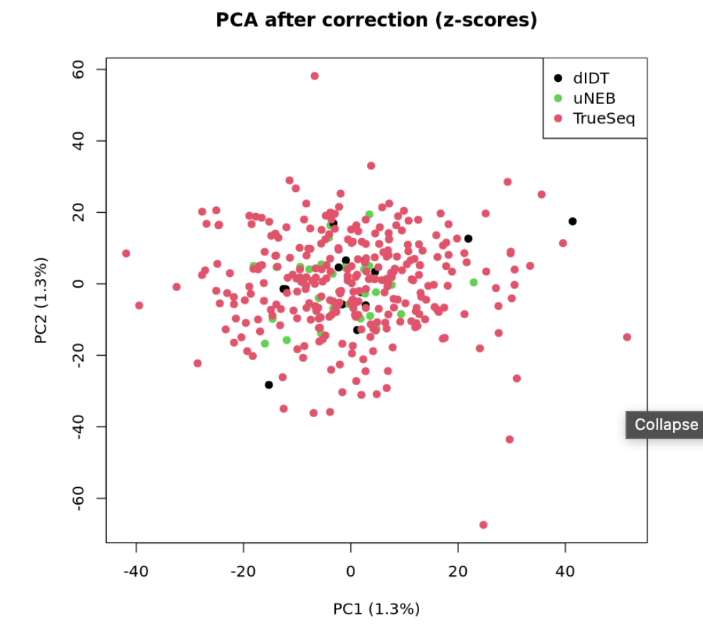


a

b

**PCA plots generated with OUTRIDER before and after noise and confounder correction**

These PCA plots show the variance between the RNA-sequencing data (raw counts) before and after noise and confounder correction. **a,** Shows the variance between the log2-transformed RNA-sequencing counts before noise and confounder correction by the autoencoder. **b,** Shows the variance between the calculated *z-*scores based on the count files after noise and confounder correction by the autoencoder. The color of the datapoints corresponds to the different RNA library preparation kits that have been used for the UMCG (reverse stranded NEBNext PolyA kit and NEBNext RNA Ultra II Directional (Illumina, Inc.), forward stranded dIDT TruSeq Compatible Plate Duplex RNA (Integrated DNA Technologies, Inc)) and the GTEX (unstranded TruSeq poly(A)+ RNA kits (Illumina, Inc.)) samples.

**
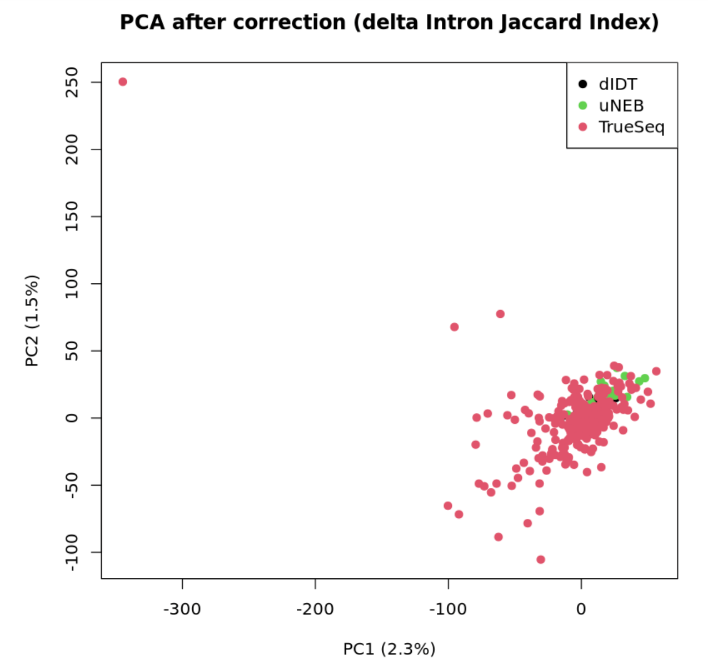

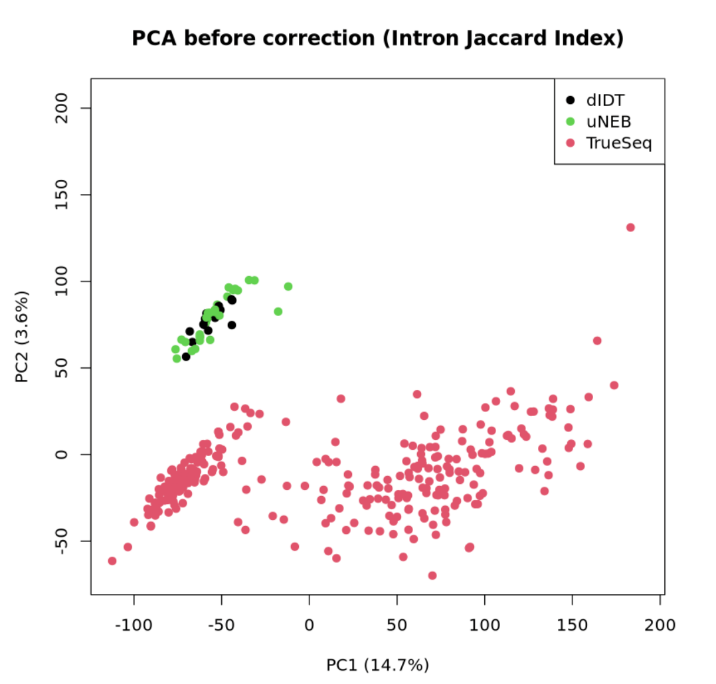
**

**PCA plots generated with FRASER before and after noise and confounder correction**

These PCA plots show the variance between the RNA-sequencing data before and after noise and confounder correction. **a,** Shows the variance between the Intron Jaccard Indices before noise and confounder correction by the autoencoder. **b,** Shows the variance between the delta Intron Jaccard Indices based on the count files after noise and confounder correction by the autoencoder. The color of the datapoints corresponds to the different RNA library preparation kits that have been used for the UMCG (reverse stranded NEBNext PolyA kit and NEBNext RNA Ultra II Directional (Illumina, Inc.), forward stranded dIDT TruSeq Compatible Plate Duplex RNA (Integrated DNA Technologies, Inc)) and the GTEX (unstranded TruSeq poly(A)+ RNA kits (Illumina, Inc.)) samples.

**References**

1. Jaramillo Oquendo C, Wai HA, Rich WI, Bunyan DJ, Thomas NS, Hunt D, Lord J, Douglas AGL, Baralle D. Identification of diagnostic candidates in Mendelian disorders using an RNA sequencing-centric approach. *Genome Med* (2024) 16:110. doi: 10.1186/s13073-024-01381-w

2. Maassen WTK, Pape CCET, Urzua-Traslavina CG, Niemeijer T, van Lieshout T, van der Molen M, Johansson LF, van der Velde KJ, Franke L, van Gijn ME, et al. Accelerating rare disease diagnostics by linking DNA and RNA through an 2 explainable and interactive RNA-guided workflow (in press). *NAR Genom Bioinform* (2026)lqag016. doi: https://doi.org/10.1093/nargab/lqag016

3. Scheller IF, Lutz K, Mertes C, Yepez VA, Gagneur J. Improved detection of aberrant splicing with FRASER 2.0 and the intron Jaccard index. *Am J Hum Genet* (2023) 110:2056–2067. doi: 10.1016/j.ajhg.2023.10.014
