# Supplementary material for: A multidisciplinary RNA-guided approach to complement genomic analysis of unsolved patients with an inborn error of immunity
Source: Front Immunol. 2026 May 28;17:1829883. doi: 10.3389/fimmu.2026.1829883 (PMC13252776; doi:10.3389/fimmu.2026.1829883)
Supplement: Supplementary Data Sheet 7 — Inconclusive cases. [file DataSheet7.docx]

## Supplementary data 7: Analysis results inconclusive cases


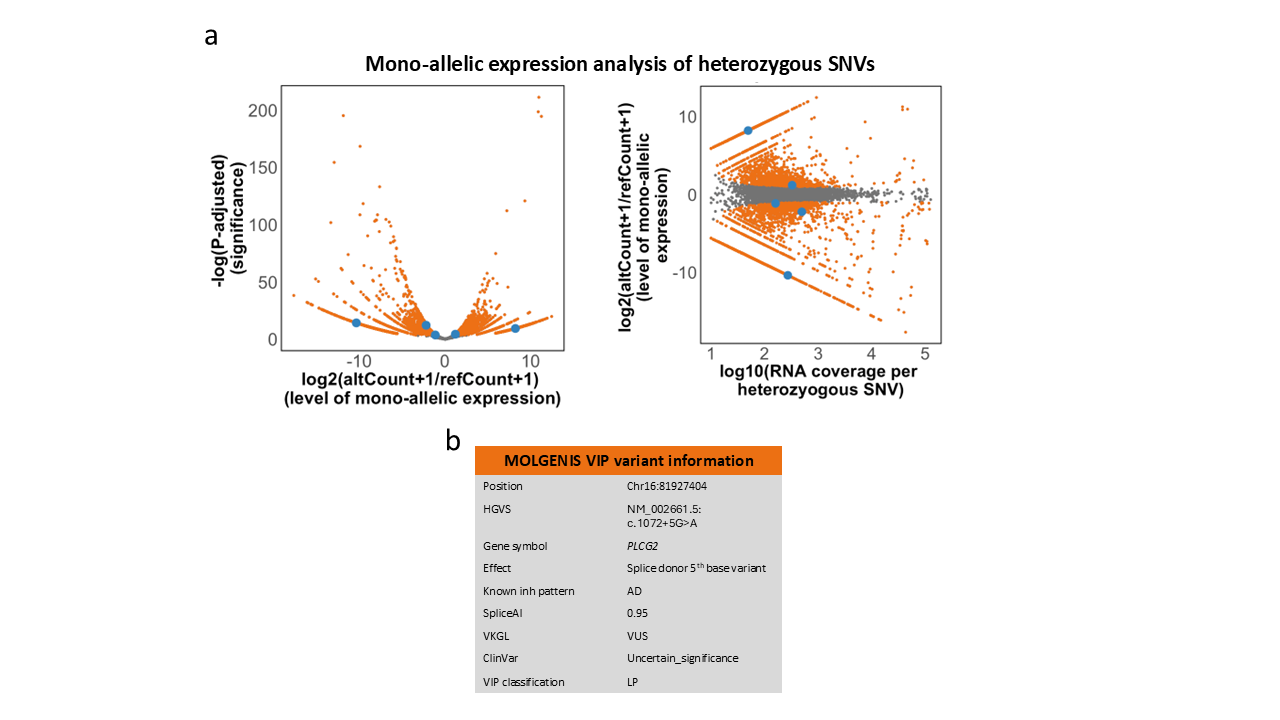
**Case: *PLCG2* (RNA_PID_007_C)**

**a,** tMAE analysis. Each dot represents a heterozygous single nucleotide variant (SNV). Blue dots represent heterozygous SNVs in *PLCG2*. Orange dots are SNVs that are significant (*P*-adjusted < 0.05). X-axis shows the log2 of the alternative allele ratios that indicates the represent level of mono-allelic expression. Y-axis shows the *p-*adjusted value representing the significance of the mono-allelically expressed heterozygous SNVs in the context of all heterozygous SNVs of per patients. **b**, tMAE analysis. Each dot represents a heterozygous SNV. Blue dots represent heterozygous SNVs in *PLCG2*. X-axis shows the log10 of the RNA-sequencing coverage per heterozygous SNV and the Y-axis shows the log2 of the alternative allele ratios that indicates the level of mono-allelic expression. **c**, VIP annotations for NM_002661.5: c.1072+5G>A.


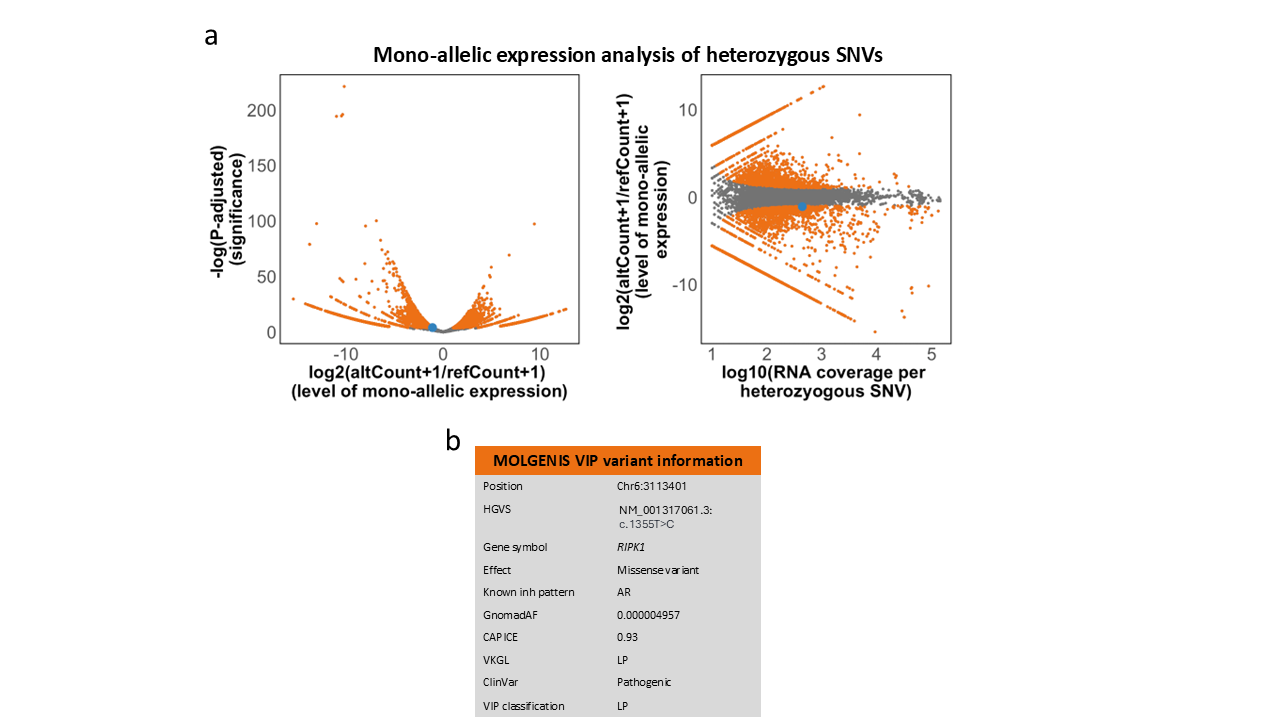
**Case: *RIPK1* (RNA_PID_004_C)**

**a,** tMAE analysis. Each dot represents a heterozygous single nucleotide variant (SNV). Blue dots represent heterozygous SNVs in *RIPK1*. Orange dots are SNVs that are significant (*P*-adjusted < 0.05). X-axis shows the log2 of the alternative allele ratios that indicates the represent level of mono-allelic expression. Y-axis shows the *p-*adjusted value representing the significance of the mono-allelically expressed heterozygous SNVs in the context of all heterozygous SNVs of per patients. **b**, tMAE analysis. Each dot represents a heterozygous SNV. Blue dots represent heterozygous SNVs in *RIPK1*. X-axis shows the log10 of the RNA-sequencing coverage per heterozygous SNV and the Y-axis shows the log2 of the alternative allele ratios that indicates the level of mono-allelic expression. **c**, VIP annotations for NM_001317061.3: c.1355T>C.


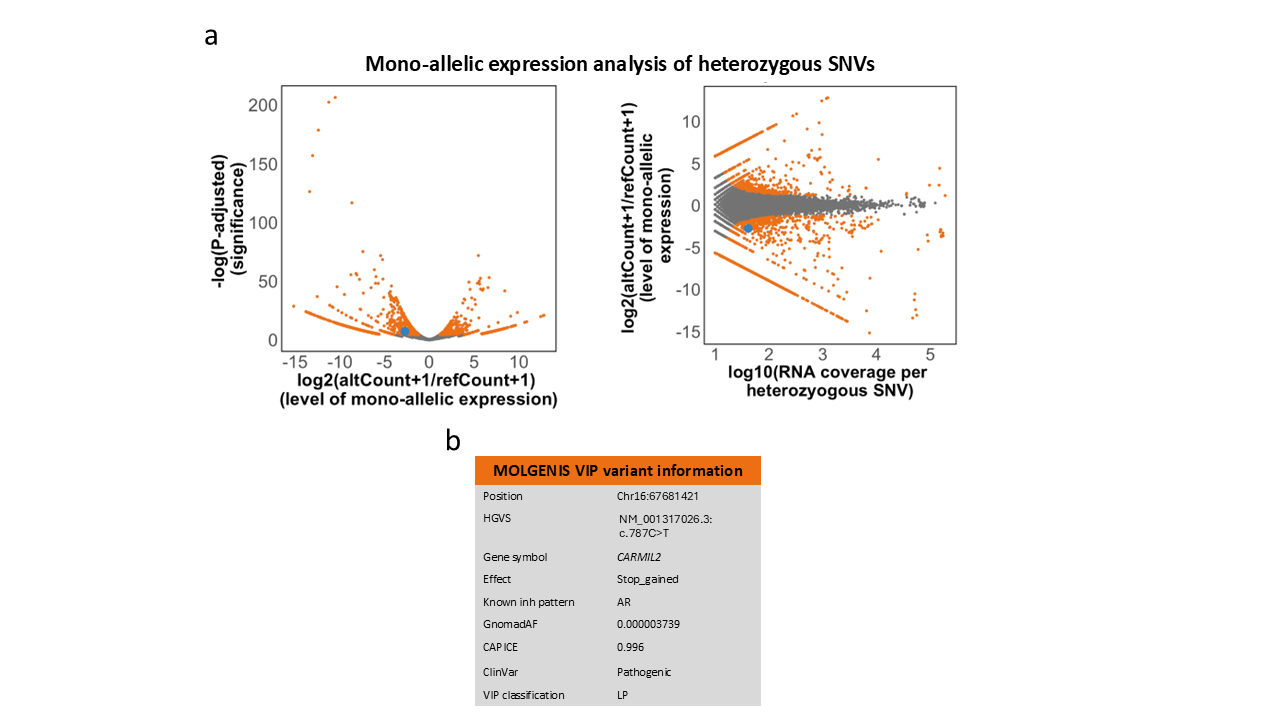
**Case: *CARMIL2* (RNA_PID_003_C)**

**a,** tMAE analysis. Each dot represents a heterozygous single nucleotide variant (SNV). Blue dots represent heterozygous SNVs in *CARMIL2*. Orange dots are SNVs that are significant (*P*-adjusted < 0.05). X-axis shows the log2 of the alternative allele ratios that indicates the represent level of mono-allelic expression. Y-axis shows the *p-*adjusted value representing the significance of the mono-allelically expressed heterozygous SNVs in the context of all heterozygous SNVs of per patients. **b**, tMAE analysis. Each dot represents a heterozygous SNV. Blue dots represent heterozygous SNVs in *CARMIL2*. X-axis shows the log10 of the RNA-sequencing coverage per heterozygous SNV and the Y-axis shows the log2 of the alternative allele ratios that indicates the level of mono-allelic expression. **c**, VIP annotations for NM_001317026.3: c.787C>T.

**Case: *NCKAP1L* (RNA_PID_012_C)**


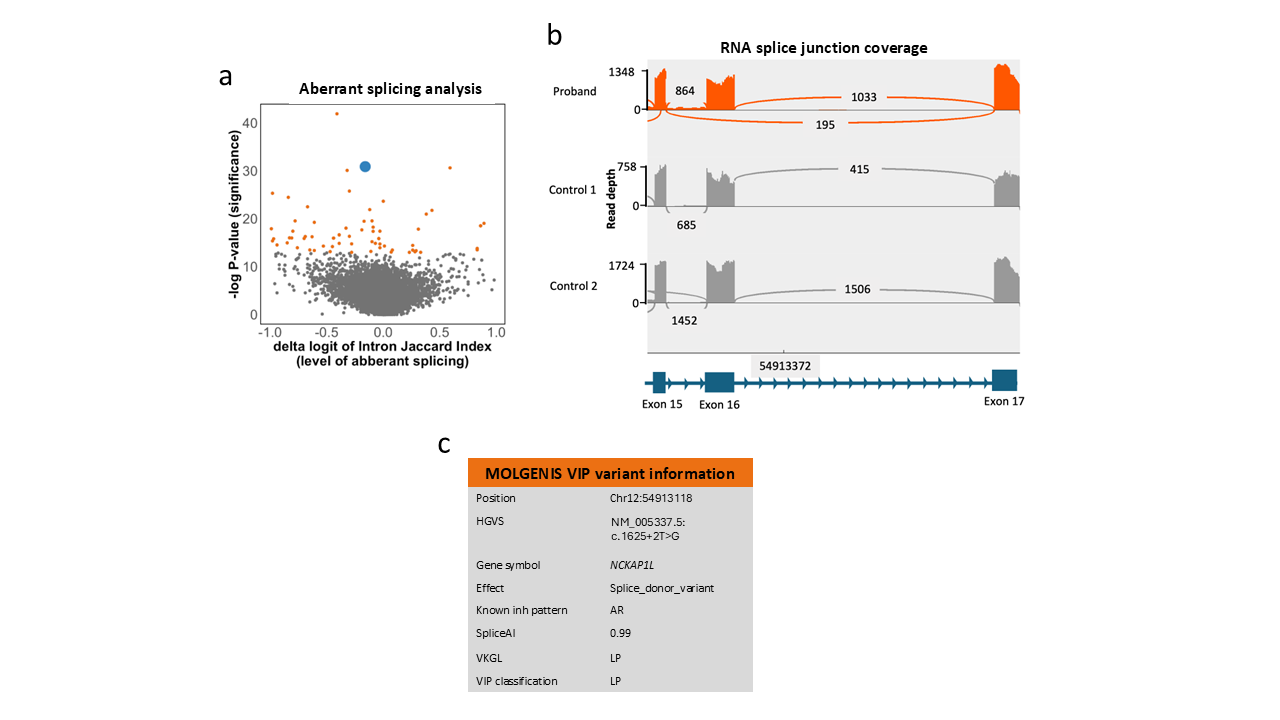


**delta-psi (level of aberrant splicing)**

**a,** Aberrant splicing analysis. Each dot represents the usage of a splice junction in the proband in each gene. X-axis shows the delta-psi value which indicates the level of aberrant splicing. Y-axis shows a -log *p*-value indicating the significance of the splicing outlier compared to the usage of splice junctions across all patients in the complete cohort. The blue dot indicates the gene, *NCKAP1L.* **b**, Sashimi plot showing the reads in RNA-seq data that cover exons 15, 16 and 17 for the patient. **c**, VIP annotations for NM_005337.5: c.1625+2T>G.

**Case: *DOCK8* (RNA_PID_022_C)**


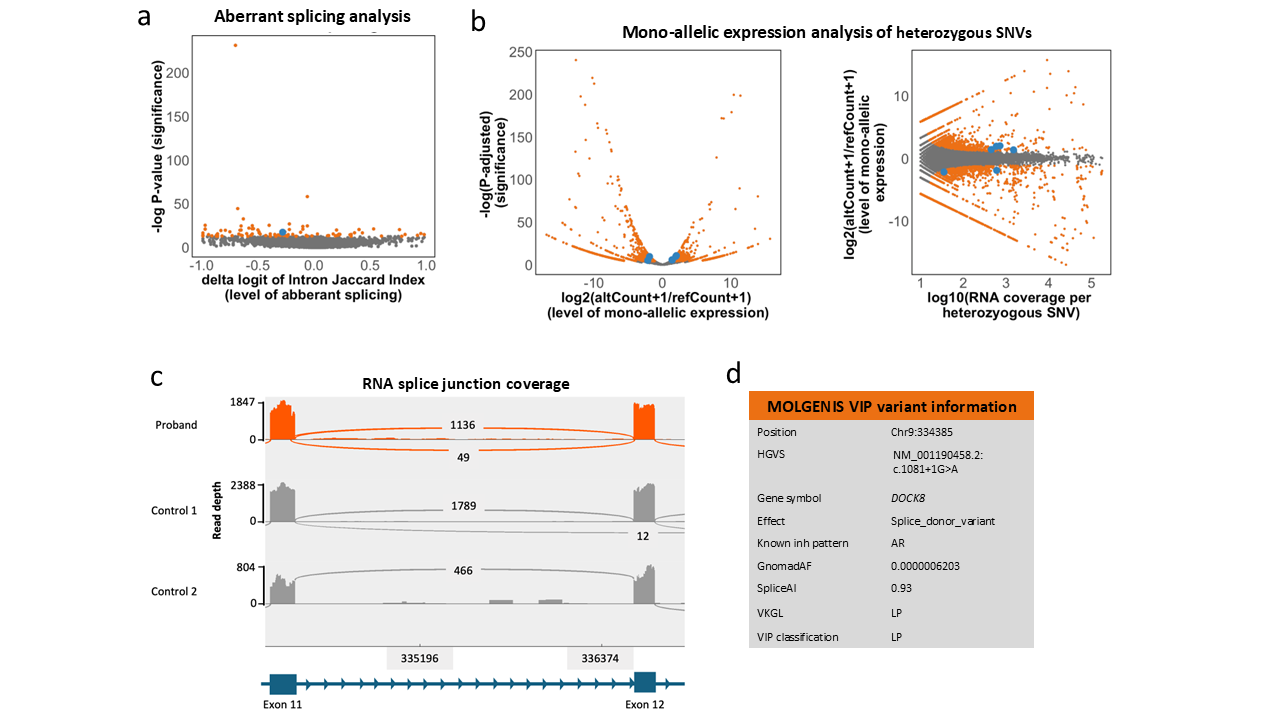


**delta-psi (level of aberrant splicing)**

**a,** Aberrant splicing analysis. Each dot represents the usage of a splice junction in the proband in each gene. X-axis shows the delta-psi value which indicates the level of aberrant splicing. Y-axis shows a -log *p*-value indicating the significance of the splicing outlier compared to the usage of splice junctions across all patients in the complete cohort. The blue dot indicates the gene, *DOCK8.* **b,** tMAE analysis. Each dot represents a heterozygous single nucleotide variant (SNV). Blue dots represent heterozygous SNVs in *DOCK8*. Orange dots are SNVs that are significant (*P*-adjusted < 0.05). X-axis shows the log2 of the alternative allele ratios that indicates the represent level of mono-allelic expression. Y-axis shows the *p-*adjusted value representing the significance of the mono-allelically expressed heterozygous SNVs in the context of all heterozygous SNVs of per patients. **b**, tMAE analysis. Each dot represents a heterozygous SNV. Blue dots represent heterozygous SNVs in *DOCK8*. X-axis shows the log10 of the RNA-sequencing coverage per heterozygous SNV and the Y-axis shows the log2 of the alternative allele ratios that indicates the level of mono-allelic expression. **c**, Sashimi plot showing the reads in RNA-seq data that cover exons 11 and 12 for the patient. **d**, VIP annotations for NM_001190458.2: c.1081+1G>A.
